# Bidirectional Effect of Long-Term Δ^9^-Tetrahydrocannabinol Treatment on mTOR Activity and Metabolome

**DOI:** 10.1021/acsptsci.4c00002

**Published:** 2024-08-14

**Authors:** Andras Bilkei-Gorzo, Britta Schurmann, Marion Schneider, Michael Kraemer, Prakash Nidadavolu, Eva C. Beins, Christa E. Müller, Mona Dvir-Ginzberg, Andreas Zimmer

**Affiliations:** † Institute of Molecular Psychiatry, Medical Faculty, University of Bonn, Bonn 53125, Germany; ‡ Pharmaceutical Institute, University of Bonn, Bonn 53121, Germany; § Institute of Forensic Medicine, Medical Faculty, University of Bonn, Bonn 53111, Germany; ∥ Institute of BioMedical and Oral Research, Faculty of Dental Medicine, Hebrew University of Jerusalem, Jerusalem 9112102, Israel

**Keywords:** antiaging, brain, mTOR, synaptic proteins, metabolome, tetrahydrocannabinol, blood plasma, adipose tissue

## Abstract

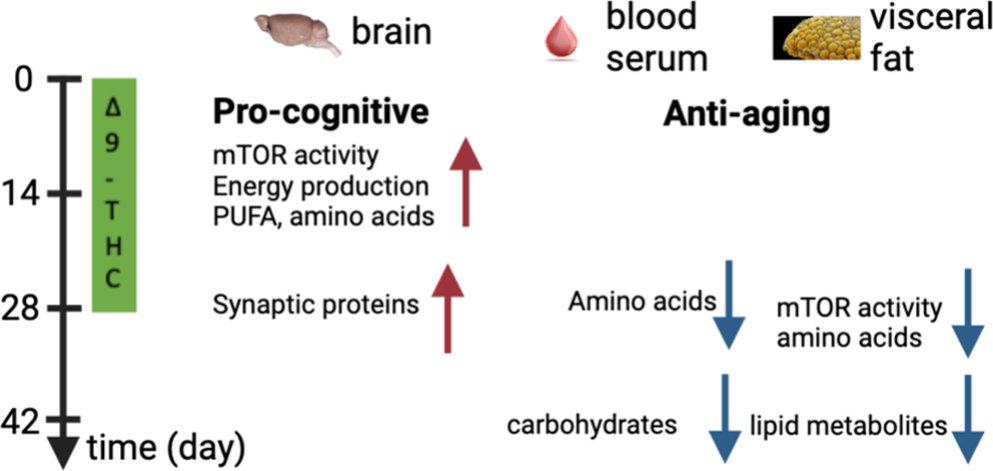

Brain aging is associated with cognitive decline, reduced synaptic plasticity, and altered metabolism. The activity of mechanistic target of rapamycin (mTOR) has a major impact on aging by regulating cellular metabolism. Although reduced mTOR signaling has a general antiaging effect, it can negatively affect the aging brain by reducing synaptogenesis and thus cognitive functions. Increased mTOR activity facilitates aging and is responsible for the amnestic effect of the cannabinoid receptor 1 agonist Δ^9^-tetrahydrocannabinol (THC) in higher doses. Long-term low-dose Δ^9^-THC had an antiaging effect on the brain by restoring cognitive abilities and synapse densities in old mice. Whether changes in mTOR signaling and metabolome are associated with its positive effects on the aging brain is an open question. Here, we show that Δ^9^-THC treatment has a tissue-dependent and dual effect on mTOR signaling and the metabolome. In the brain, Δ^9^-THC treatment induced a transient increase in mTOR activity and in the levels of amino acids and metabolites involved in energy production, followed by an increased synthesis of synaptic proteins. Unexpectedly, we found a similar reduction in the mTOR activity in adipose tissue and in the level of amino acids and carbohydrate metabolites in blood plasma as in animals on a low-calorie diet. Thus, long-term Δ^9^-THC treatment first increases the level of energy and synaptic protein production in the brain, followed by a reduction in mTOR activity and metabolic processes in the periphery. Our study suggests that a dual effect on mTOR activity and the metabolome could be the basis for an effective antiaging and pro-cognitive medication.

The cannabinoid receptor type-1 (CB_1_) is the most prominent G-protein-coupled receptor in the brain. Its expression level is also high in several peripheral organs like the liver, adipose tissue, and muscle.^1^ CB_1_ can be activated by the ligands *N*-arachidonoylethanolamine (AEA, anandamide) and 2-arachidonoylglycerol (2-AG), as well as by the phytocannabinoid Δ^9^-tetrahydrocannabinol (Δ^9^-THC) and synthetic compounds.

Previous studies indicated a link between CB_1_ receptor activity and brain aging. Thus, Cnr1^–/–^ mice, which have a genetic deletion of CB_1_ receptors, showed enhanced age-related deficits in learning and memory accompanied by a loss of neurons, reduced neurogenesis, and concomitantly enhanced signs of neuroinflammation.^2^ Elevation of CB_1_ receptor activity had the opposite effect: It alleviated several symptoms of brain aging and restored cognitive functions in older mice.^3^ The long-lasting cognitive improvements in Δ^9^-THC-treated old mice were associated with hippocampal transcriptional profiles that were reminiscent of those of young animals, which lasted for several weeks after cessation of the treatment. On a cellular level, we observed enhanced synaptic protein synthesis, as well as an increased hippocampal dendritic spine density.^3^ In the cortex, a similar treatment of old mice improved dendritic spine stability, also leading to a long-lasting increase in spine density.^4^ It is known that dynamic changes in dendritic spines are involved in synaptic plasticity, and thus contribute to learning and memory formation.^5^ We propose that the Δ^9^-THC treatment-induced increase in synapses underlies its effect against brain aging in old mice.

Numerous studies indicated that CB_1_ receptor activity also influences critical aging-associated metabolic processes like proteostasis, nutrient sensing, and mitochondrial activity.^6,7^ Some of these effects may be mediated directly by mitochondrial CB_1_ receptors or indirectly through cell membrane-associated receptors. Activation of mitochondrial CB_1_ decreases oxygen consumption, reactive oxygen species (ROS) production,^8^ and oxidative phosphorylation.^9^ Moreover, as activation of neuronal surface CB_1_ receptors decreases firing frequency, and activation of mitochondrial CB_1_ receptors decreases mitochondrial activity, it enables a coupling between firing activity and energy need of the neurons.^10^ Several studies demonstrate that both the CB_1_ receptor agonist HU210^11^ and genetic variance in genes encoding elements of cannabinoid system^12^ influence BOLD signal intensities in the brain, which further support the possible role of cannabinoid signaling in brain energetics.

Synaptic plasticity is largely dependent on dynamic changes in the strength and number of connections between neurons. De novo local lipid and protein synthesis plays a crucial role in it, which is regulated by the mechanistic target of rapamycin (mTORC1) signaling.^13^ Activity of mTORC1 affects cellular energy balance by coupling the availability of nutrients and growth factor signaling with energy-demanding functions like lipid and protein biosynthesis.^14^ A tight coordination of these processes is necessary for the formation of new synapses. Increased levels of growth factors and/or nutrients lead to a phosphorylation of mTORC1, which blocks autophagy and stimulates protein synthesis. Importantly, alteration in mTORC1 signaling can stimulate or suppress learning and memory, probably depending on the duration and level of its activation.^15^ Blockade of mTORC1 by rapamycin treatment impairs consolidation of long-term memories, whereas increasing mTORC1 activity—to an extent—improves memory.^16^ It is known that Δ^9^-THC increases mTORC1 activity, which contributes to the amnesic effect of acute high dose Δ^9^-THC treatment.^17^ Whether altered mTORC1 signaling is associated with the positive effects of long-term low-dose Δ^9^-THC on the aged brain is an open question.

Both acute and long-term Δ^9^-THC treatment has a significant effect on the metabolome: acute Δ^9^-THC in higher dose decreases brain glucose metabolism,^10,18^ whereas long-term low-dose treatment improved brain energetics in rodents^19^ and human^20^ alike. Whether the positive effect of long-term low-dose Δ^9^-THC on brain aging is associated with changes in the brain and body metabolome is yet unknown.

## Results

### Effect of Δ^9^-THC Treatment on Body Weight, Food Intake, and Motility

Changes in body composition, food intake, or activity all can have strong effects on the metabolome; therefore, we first asked whether Δ^9^-THC treatment or withdrawal influenced these parameters. As shown in Figure S1A, body weight change did not differ between vehicle and Δ^9^-THC-treated mice. The loss of body weight at day 3 is likely the result of the stress associated with the surgical implantation of the minipumps at day 0. Although the body weight changes did not differ between the groups, Δ^9^-THC-treated mice ate less at the very end of the treatment period (treatment × time interaction: *F*_12,408_ = 2.613; *p* = 0.0023) (Figure S1B). The home cage activity and also the diurnal cycle were indistinguishable between vehicle and Δ^9^-THC-treated mice during the last 90 h of Δ^9^-THC treatment (treatment effect: *F*_1,18_ = 0.344; *p* = 0.5658) or the last 42 h of the withdrawal phase (treatment effect: *F*_1,16_ = 1.009; *p* = 0.3302) (Figure S1C).

### Long-Term Low-Dose Δ^9^-THC Treatment Leads to a Temporary Increase in mTOR Phosphorylation in the Brain

**Figure 1:**
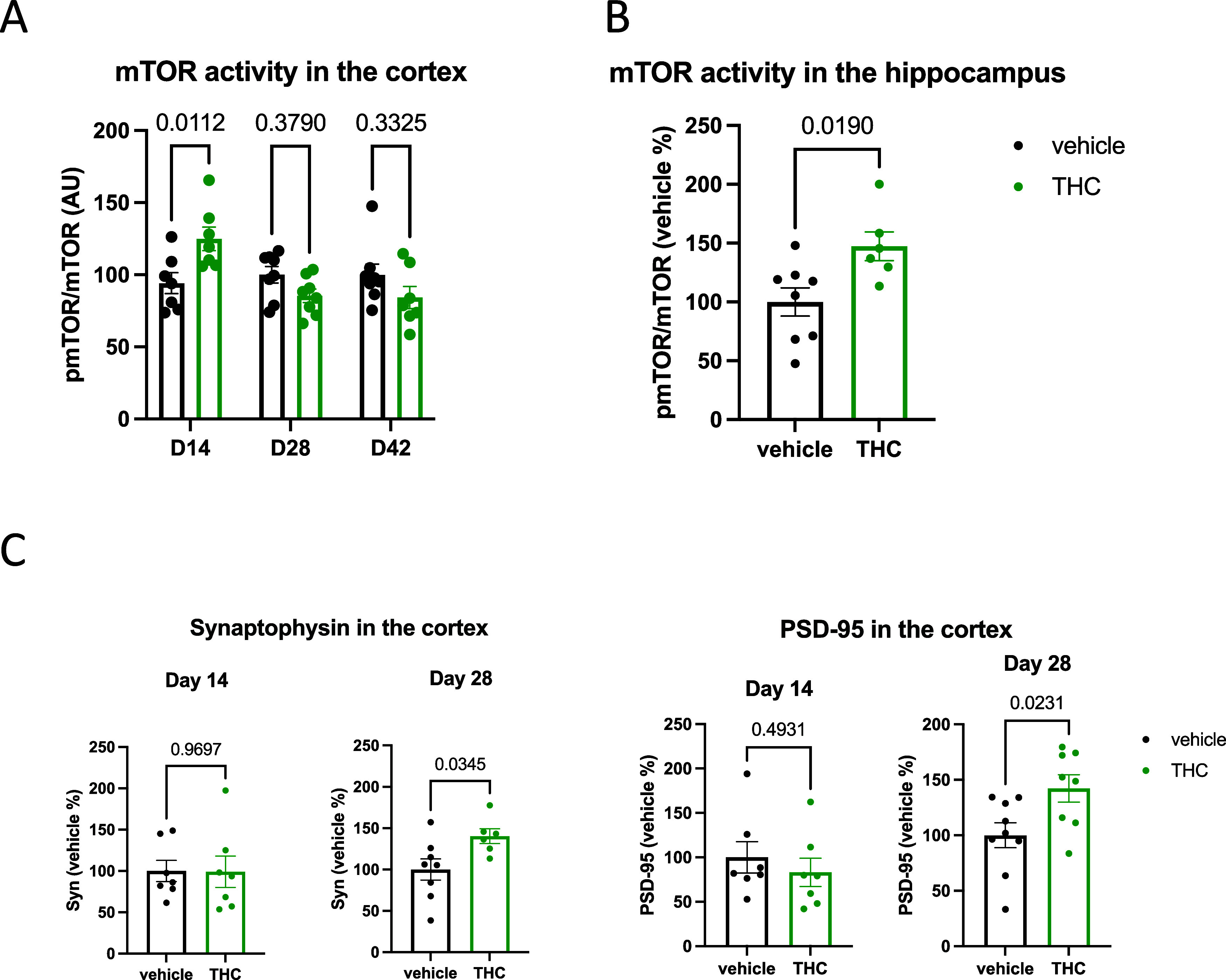
(A) Long-term low-dose THC treatment leads to a temporary increase in mTOR phosphorylation in the cortex. Significances were calculated by Bonferroni’s *t* test after two-way ANOVA. (B) Enhanced mTOR phosphorylation in the hippocampus of THC-treated animals at day 14. Significance is calculated by Student’s unpaired *t* test. (C) Increased synaptophysin and PSD-95 levels in the cortex of THC-treated animals at day 28. Significances are calculated by Student’s unpaired *t* test. Columns represent mean values, whiskers represent standard error of mean (SEM), and dots represent the individual values.

We compared mTOR levels and phosphorylation ratios in the cortex of mice after 14 and 28 days of Δ^9^-THC treatment and at day 42, after 14 days of withdrawal. The treatment did not change mTOR levels (*F*_1,41_ = 0.489; *p* = 0.489), and we found no treatment × time interaction (*F*_2,41_ = 0.491; *p* = 0.615). The activity of mTOR, i.e., its phosphorylation level, however, was temporarily altered in Δ^9^-THC-treated animals shown by the significant treatment × time interaction (*F*_2,39_ = 7.302; *p* = 0.002). Indeed, post hoc analysis revealed that mTOR activity was significantly higher at day 14 in Δ^9^-THC-treated mice (Figure 1A). Additionally, we tested whether mTOR activity was also increased in the hippocampus after 14 days of Δ^9^-THC treatment using a separate animal cohort. Indeed, we found a similarly increased phosphorylation of mTOR in the hippocampi of Δ^9^-THC-treated mice at day 14 (+47.30%; *t*_12_ = 2.708; *p* = 0.0190; Figure 1B).

Next, we wanted to know whether Δ^9^-THC treatment increased the amount of synaptic proteins in the cortex, similarly to our previous observation in the hippocampus.^3^ Interestingly, the amount of synaptophysin (+40.2%; *t*_12_ = 2.384; *p* = 0.0345) and PSD-95 (+42.2%; *t*_15_ = 2.530; *p* = 0.0231) was higher in Δ^9^-THC-treated animals than in controls at day 28, but not at day 14 (−14.53%; *t*_15_ = 0.981; *p* = 0.3421 for synaptophysin and +16.4%; *t*_14_ = 0.7131; *p* = 0.4874 for PSD-95), at the time of peak mTOR activity (Figure 1C).

### Metabolome Signatures of Enhanced Energy Levels and Synthetic Activity at Day 14 in the Hippocampus of Δ^9^-THC-Treated Animals

**Figure 2:**
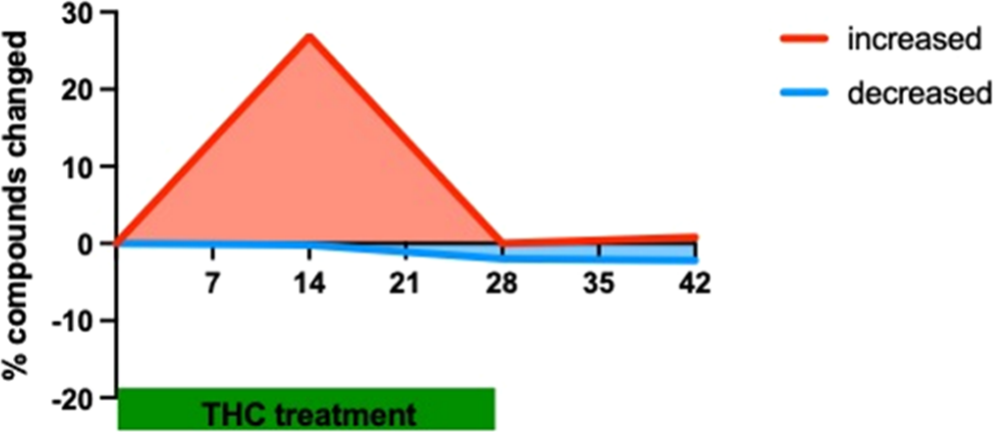
Percentage of significantly changed metabolites according to two-way ANOVA and Welch’s two-sample *t* tests in the hippocampus during and after THC treatment.

In the hippocampus, long-term Δ^9^-THC treatment for 14 days induced a significant increase in the level of 109 from the totally measured 494 metabolites (22%), an effect that mostly disappeared at day 28 (Figure 2).

**Figure 3:**
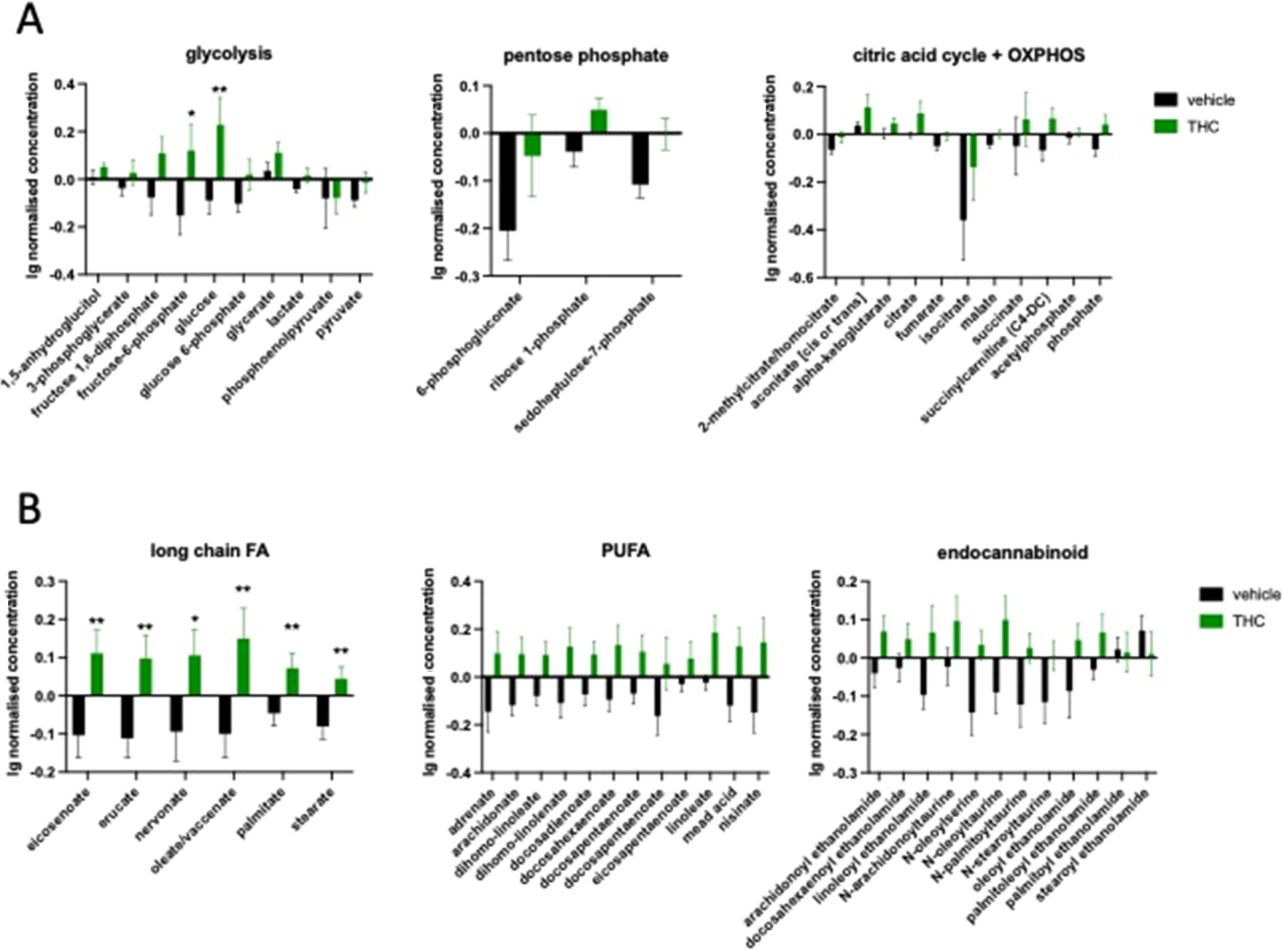
(A) THC treatment increases the level of compounds representing carbohydrate metabolism and energy production at day 14 in the hippocampus. OXPHOS = oxidative phosphorylation. (B) Significant increase in the level of lipid metabolites with known antiaging effect. FA = fatty acid; PUFA = polyunsaturated fatty acid. Significantly affected compound groups were identified using two-way repeated ANOVA. Columns represent mean values, whiskers standard error. *q < 0.05; **q < 0.01 difference between the vehicle- and THC-treated groups according to Benjamin, Krieger, and Yekutieli.

The 14 days of withdrawal also did not induce major changes in the metabolite levels (Figure 2); therefore, we focused our further analysis on the D14 time point. We found a significant increase in the level of most of the metabolite groups representing carbohydrate metabolism and energy production: in glycolysis (treatment effect: *F*_1,14_ = 6.815; *p* = 0.0206), and pentose phosphate metabolism (treatment effect: *F*_1,14_ = 6.951; *p* = 0.0195) as well as in metabolites of the citric acid cycle and oxidative phosphorylation (treatment effect: *F*_1,14_ = 5.213; *p* = 0.0386) (Figure 3A).

Additionally, we found an enhanced metabolism of two other groups of carbohydrates in Δ^9^-THC-treated mice, namely, in pentose metabolism (treatment effect: *F*_1,14_ = 6.524; *p* = 0.0229) and fructose metabolism (treatment effect: *F*_1,14_ = 7.467; *p* = 0.0162) (Table S1). Δ^9^-THC treatment also induced a significant increase in the level of several lipid metabolites with known antiaging effect, including long-chain fatty acids (treatment effect: *F*_1,14_ = 5.939; *p* = 0.0288), polyunsaturated fatty acids (treatment effect: *F*_1,14_ = 5.351; *p* = 0.0364), as well as endocannabinoids (compound × treatment: *F*_12,168_ = 2.065; *p* = 0.0217) (Figure 3B). Interestingly, the level of monoacylglycerols (treatment effect: *F*_1,14_ = 5.207; *p* = 0.0387) including the major endogenous CB_1_ receptor agonist 2-arachidonoylglycerol and several members of the lysophospholipids (compound × treatment: *F*_22,308_ = 2.637; *p* > 0.0001) were also enhanced in Δ^9^-THC-treated animals (Table S1). Among amino acids, the concentration of glutamate (treatment effect: *F*_1,14_ = 4.642; *p* = 0.0491) and all of the proteinogenic branched-chain amino acid—leucine, isoleucine, and valine—metabolites were upregulated in Δ^9^-THC-treated mice (treatment effect: *F*_1,14_ = 6.207; *p* = 0.0259) (Table S1).

### Consecutive Bidirectional Change in Blood Plasma Metabolome

**Figure 4:**
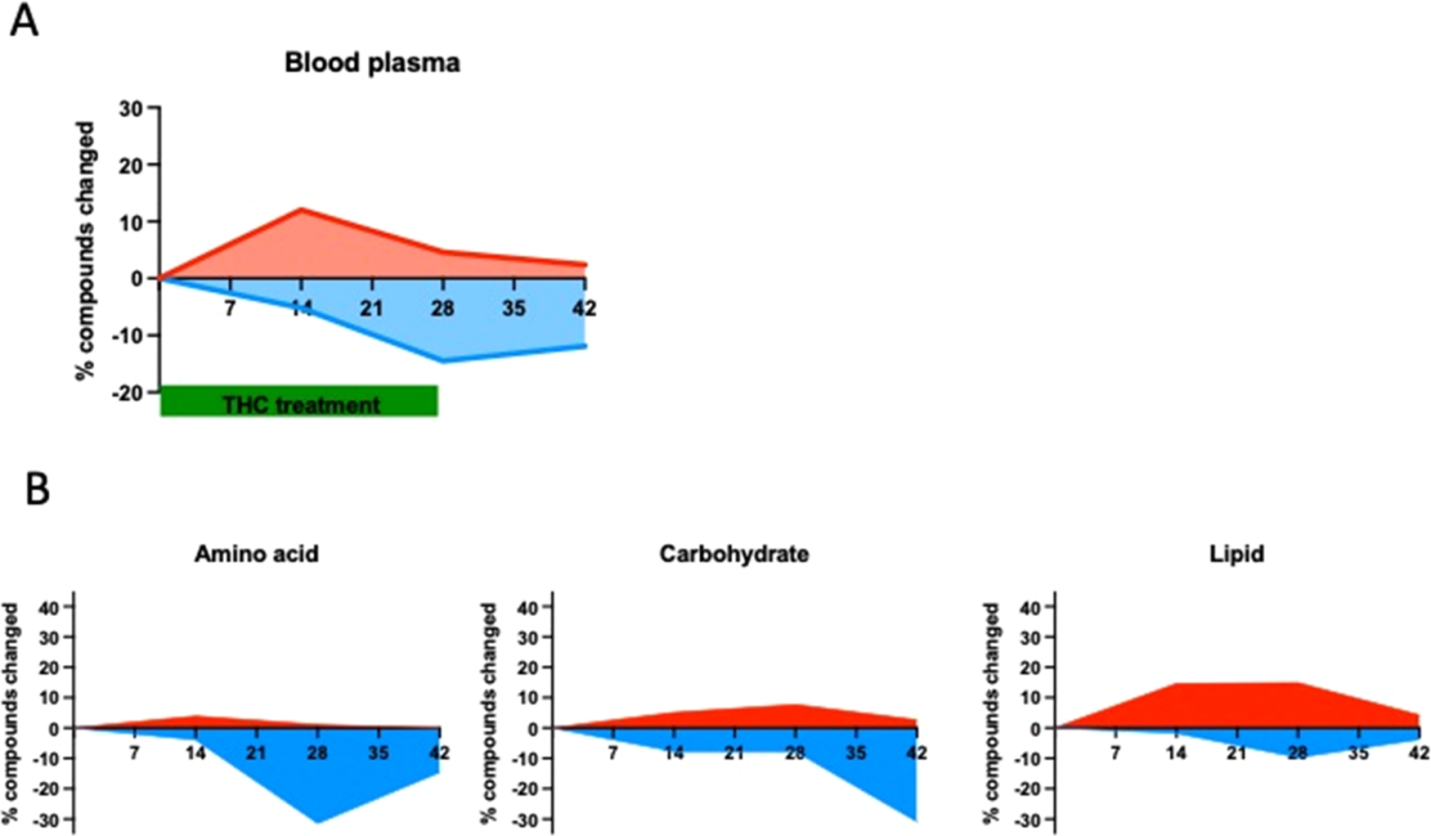
(A) Percentage of significantly up- and downregulated metabolites in the blood plasma during and after THC treatment. (B) Percentage of significantly changed amino acids, carbohydrates, and lipids metabolites in the blood plasma during and after THC treatment. The identification of compounds with significantly changed levels (A) and (B) was done using two-way ANOVA followed by Welch’s two-sample *t* tests.

The Δ^9^-THC-induced changes in the plasma metabolome were highly dependent on the duration of the treatment, similar to those observed in the hippocampus. Fourteen days of treatment led to a general upregulation of metabolite concentrations (Figure 4A). Similar to the case of the hippocampus, this effect had almost disappeared by day 28. At this time point and also at day 42, after 14 days of withdrawal, the dominant change was a decline (Figure 4A).

The majority of the upregulated metabolites at day 14 were lipids (Figure 4B).

The increase was significant for fatty acid amides (treatment effect: *F*_1,14_ = 5.653; *p* = 0.0322), phosphatidylethanolamines (treatment effect: *F*_1,14_ = 11.99; *p* = 0.0038), phosphatidylinositols (treatment effect: *F*_1,14_ = 7.359; *p* = 0.0168), diacylglycerols (treatment effect: *F*_1,14_ = 10.33; *p* = 0.0062), and ceramides (treatment effect: *F*_1,14_ = 5.948; *p* = 0.0287) (Table S2). The only nonlipid metabolite group significantly affected by the Δ^9^-THC treatment at day 14 was the (hypo)xanthine/inosine containing purine metabolites (treatment effect: *F*_1,14_ = 6.954; *p* = 0.0195), whose levels was decreased (Table S2).

**Figure 5:**
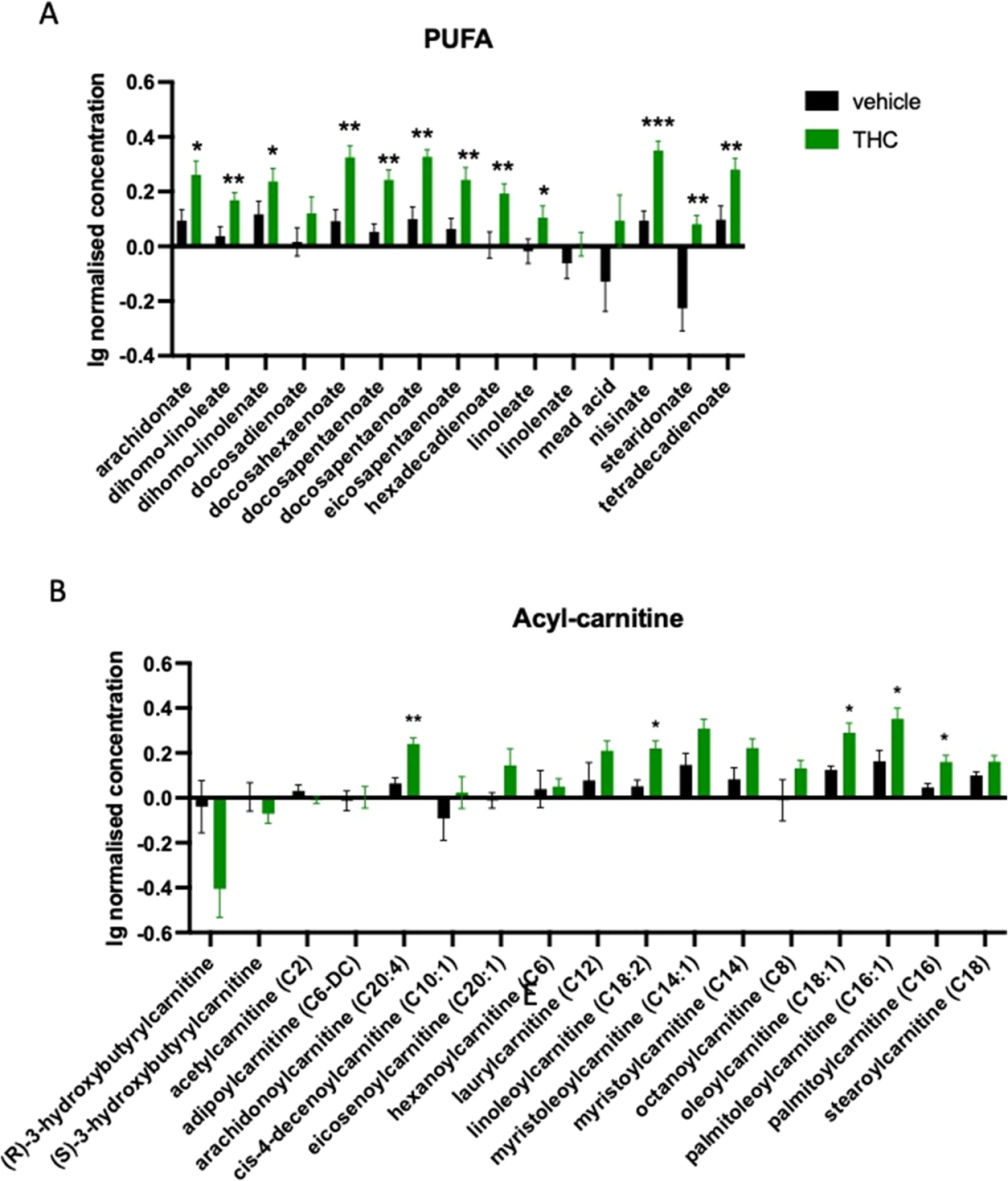
(A) Significant increase in the level of polyunsaturated fatty acids (PUFA). (B) Significant increase in the concentration of several members of the acyl-carnitines in the blood plasma of Δ^9^-THC-treated old animals. Columns represent mean values, whiskers standard error. *q < 0.05; **q < 0.01; ***q < 0.001 difference between the treatment groups according to Benjamin, Krieger, and Yekutieli.

At the end of the Δ^9^-THC treatment at day 28, only two classes of fatty acid metabolites had a higher concentration in Δ^9^-THC than in vehicle-treated animals: Polyunsaturated fatty acids (treatment effect: *F*_1,14_ = 16.52; *p* = 0.0012)—among others, the level of omega-3 long-chain polyunsaturated fatty acids docosahexaenoate (DHA), nisinate and stearidonate (Figure 5A) and several members of the acyl-carnitines (compound × treatment: *F*_16,224_ = 4.614; *p* < 0.0001) showed increased levels in the treated group (Figure 5B).

At day 28, amino acid metabolite levels typically decreased (Figure 4B). These included branched-chain amino acids (treatment effect: *F*_1,14_ = 7.761; *p* = 0.0146), lysine (treatment effect: *F*_1,14_ = 11.901; *p* = 0.0039), several methionine metabolites (compound × treatment: *F*_15,210_ = 3.457; *p* < 0.0001), phenylalanine (treatment effect: *F*_1,14_ = 17.48; *p* = 0.0009) and tryptophan (treatment effect: *F*_1,14_ = 10.91; *p* = 0.0052). Furthermore, a decline in γ-glutamyl-containing branched dipeptides was detected (compound × treatment: *F*_15,210_ = 2.330; *p* = 0.00432) (Table S3).

**Figure 6:**
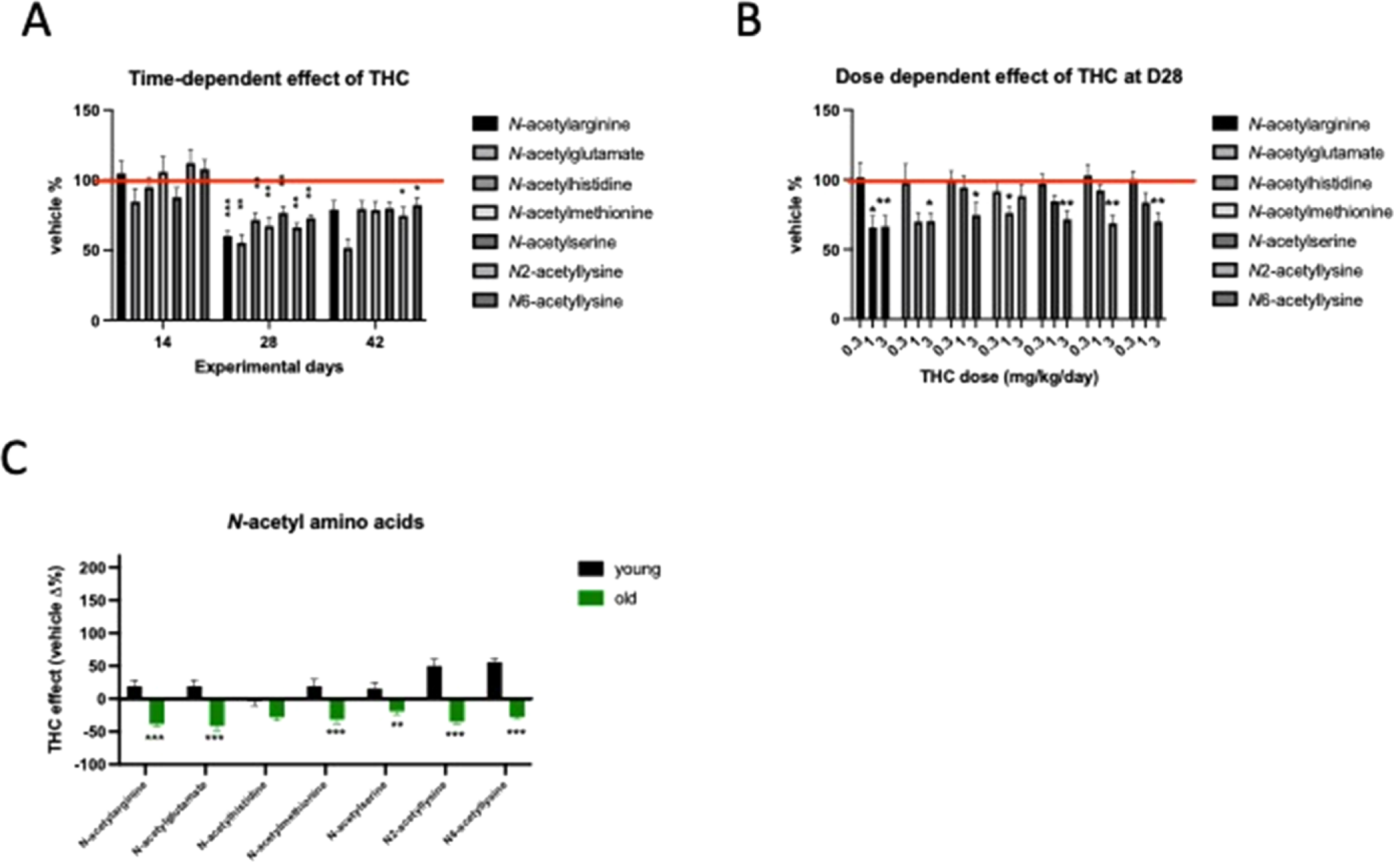
(A) Time-dependent decrease in *N*-acetylamino acid levels in the blood plasma of THC-treated animals. (B) Dose-dependent decline of *N*-acetylamino acids in the blood plasma of THC-treated animals. (C) Opposite change in *N*-acetylamino acid metabolite levels in the blood plasma of THC-treated young (4-month-old) and old (18-month-old) animals at day 28. Columns represent mean values, whiskers standard error. *q < 0.05; **q < 0.01; ***q < 0.001 difference between (A and B) vehicle and THC-treated or (C) age groups according two-way ANOVA followed by Bonferroni *t* test. Analysis was done separately for the time points (A) or compounds (B and C). Red line represents control (vehicle-treated) levels.

We realized that Δ^9^-THC treatment induced in the blood plasma the most remarkable change in the level of N-acetylated amino acids at day 28 (treatment effect: *F*_1,14_ = 34.68; *p* < 0.0001) (Figure 6A). We thus hypothesized that this could be an indicator of the metabolic reactivity to long-term Δ^9^-THC treatment. To test whether the decrease in N-acetylated amino acid levels was related to the Δ^9^-THC dose, we treated a separate group of animals with three different doses of Δ^9^-THC. As shown in Figure 6B, the Δ^9^-THC-induced decline in *N*-acetylamino acid levels was clearly dose-dependent (dose effect: *F*_2,40_ = 5.730; *p* = 0.0065).

At day 42, after 14 days of withdrawal, 11.85% of the measured 675 compounds showed reduced and only 2.37% increased plasma levels (Figure 4B). Interestingly, we could identify only two groups of compounds that were significantly influenced by the Δ^9^-THC treatment: the plasma concentrations of alanine and aspartate (treatment effect: *F*_1,14_ = 5.973; *p* = 0.0284) as well as phospholipid metabolites (treatment effect: *F*_1,14_ = 4.950; *p* = 0.0430), which were significantly lower in Δ^9^-THC-treated animals than in vehicle-treated animals (Table S4).

Lastly, we wanted to test if the prominent changes in plasma metabolite levels were dependent on the age of the animals. Thus, we treated a separate group of 4-months-old (referred as “young”) mice for 28 days and compared the Δ^9^-THC-induced changes in plasma metabolite levels with changes in the 18-month-old age group (referred as “old”). For this, we focused on metabolite classes that were altered in old animals after 28 days of Δ^9^-THC treatment. The only similarity in terms of Δ^9^-THC effects in both age groups was the increase in the concentration of polyunsaturated fatty acids (age effect: *F*_1,210_ = 0.254; *p* = 0.615) (Figure S2A). The effect of Δ^9^-THC treatment on the concentration of all other Δ^9^-THC-affected compound classes substantially differed between young and old mice: the age effect for acyl-carnitine: *F*_1,238_ = 38.47; *p* < 0.0001 (Figure S2B), branched-chain amino acids: *F*_1,392_ = 189.9; *p* < 0.0001 (Figure S3A); lysine: *F*_1,182_ = 125.6; *p* < 0.0001 (Figure S3B), methionine: *F*_1,238_ = 37.55; *p* < 0.0001 (Figure S4A), phenylalanine: *F*_1,70_ = 117.5; *p* < 0.0001 (Figure S4B), as well as tryptophan: *F*_1,280_ = 135.5; *p* < 0.0001 (Figure S5A) and the γ-glutamyl-containing branched dipeptides: *F*_1,224_ = 89.31; *p* < 0.0001 (Figure S5B). In most cases, the levels of amino acid metabolites changed in the opposite direction in Δ^9^-THC-treated young and old animals. We also detected the opposite effect of Δ^9^-THC treatment on the blood plasma levels of *N*-acetylamino acids in young and old mice (age effect: *F*_1,98_ = 219.6; *p* < 0.0001 (Figure 6C)).

### Long-Term Low-Dose Δ^9^-THC Treatment Reduced mTOR Phosphorylation in Adipose Tissue

**Figure 7:**
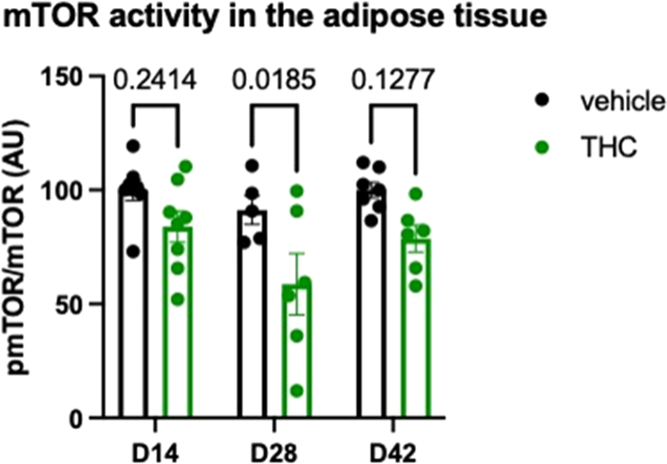
THC treatment leads to a decrease in the level of mTOR phosphorylation in the adipose tissue. Significances were calculated by Bonferroni’s *t* test after two-way ANOVA.

The activity of mTOR signaling in visceral adipose tissue was strongly reduced by the Δ^9^-THC treatment (treatment effect: *F*_1,34_ = 15.64; *p* = 0.0004). There was no treatment × time interaction (*F*_2,34_ = 0.648; *p* = 0.530), suggesting that in the adipose tissue—unlike in the cortex—the duration of the treatment or withdrawal did not alter the effect of Δ^9^-THC on mTOR phosphorylation. As shown in Figure 7, post hoc analysis of the data revealed that the decline in mTOR activity reached the level of significance at day 28, the last day of the treatment.

### Effect of Δ^9^-THC on Adipose Tissue Metabolome

**Figure 8:**
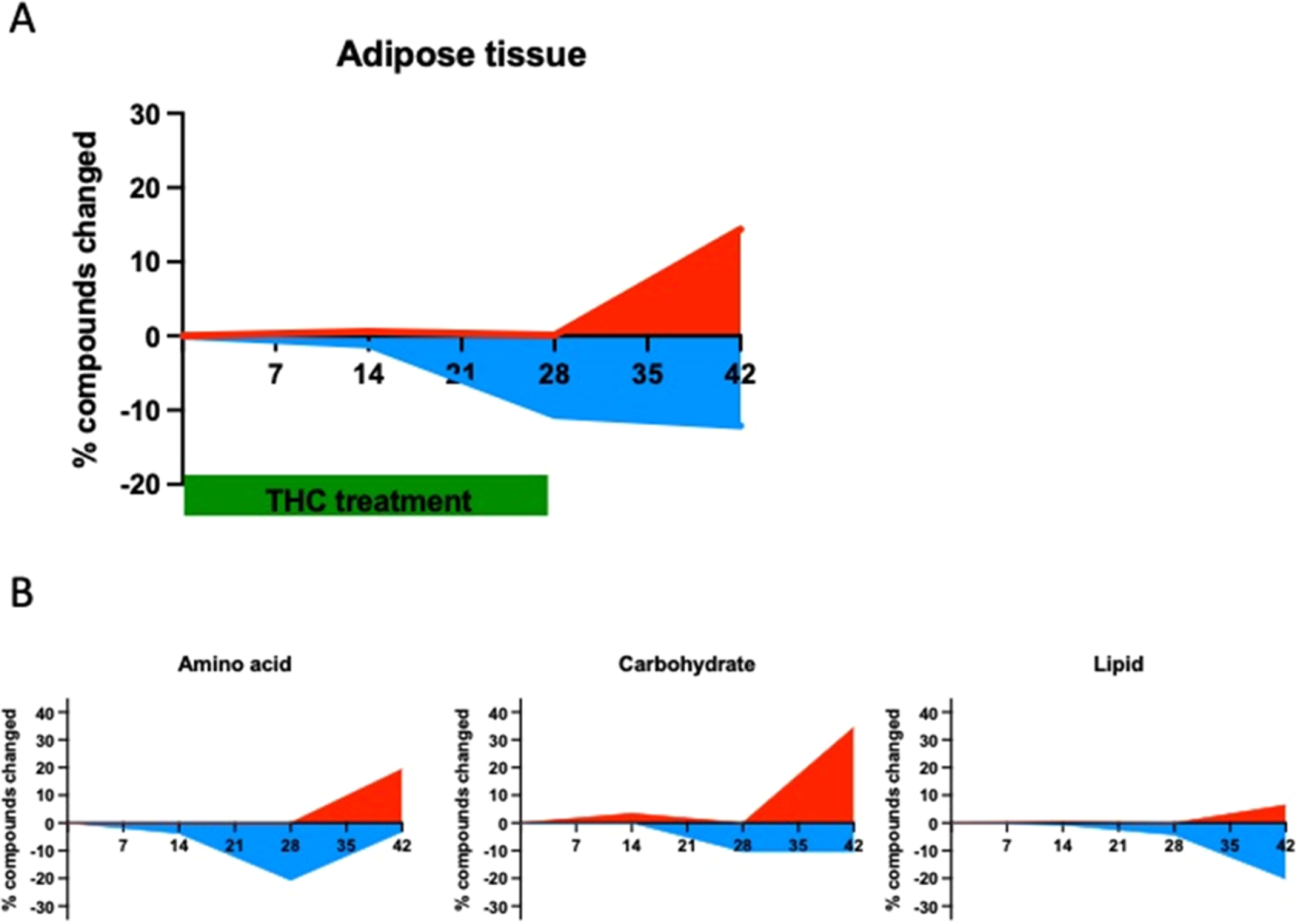
(A) Percentage of significantly up- and downregulated metabolites in the adipose tissue during and after THC treatment. (B) Percentage of significantly changed amino acids, carbohydrates, and lipids metabolites in the adipose tissue during and after THC treatment. The identification of compounds with significantly changed levels in panels (A) and (B) was done using two-way ANOVA followed by Welch’s two-sample *t* tests.

In adipose tissue, the course of changes in the three phases of the experiments was just the opposite compared to the effects observed in the brain: no changes at all on day 14, some changes (almost exclusively downregulation) on day 28, and the majority of the changes were observed in the withdrawal phase (Figure 8A). Importantly, the major metabolite groups reacted differently to Δ^9^-THC treatment in the course of the experiment: on day 28, 20.6% of the amino acid and 10.3% of the carbohydrate metabolites showed a decreased level, whereas none displayed increased levels in Δ^9^-THC-treated animals (Figure 8B). On day 42, at the 14th day of withdrawal, only 3.1% of the amino acid metabolite levels were decreased, whereas 19.4% were increased. Carbohydrates showed a similar, but more pronounced, difference at day 42: 10.3% of them had a lower, and 34.5% a higher level in the Δ^9^-THC-treated group. The levels of lipid metabolites showed little or no changes during the Δ^9^-THC treatment, whereas in the withdrawal phase, 20.2% of lipid metabolites had a lower level and 6.5% had a higher level in the Δ^9^-THC-treated group (Figure 8B).

Interestingly, our detailed analysis of the results of day 28 could identify besides one amino acid group phenylalanine (treatment effect: *F*_1,14_ = 4.761; *p* = 0.0467) only polyamines (treatment effect: *F*_1,14_ = 4.975; *p* = 0.0444) and a nucleic acid group metabolite group, orotate (treatment effect: *F*_1,14_ = 6.048; *p* = 0.0275), whose level significantly decreased. Fourteen days of withdrawal induced an increase in the level of lysine (treatment effect: *F*_1,14_ = 10.14; *p* = 0.0066), taurine (treatment effect: *F*_1,14_ = 7.288; *p* = 0.0173), and creatine (treatment effect: *F*_1,14_ = 8.267; *p* = 0.0122). Importantly, we found clear signs of a change in energy expenditure because the level of glycogens strongly decreased (treatment effect: *F*_1,14_ = 17.51; *p* = 0.0009), whereas the level of pentose metabolites increased (treatment effect: *F*_1,14_ = 11.32; *p* = 0.0046). At day 42, the concentration of lipids was most significantly influenced in the adipose tissue: the level of carnitine and acyl-carnitines, indicative of lipid metabolism, was increased (treatment effect: *F*_1,14_ = 8.004; *p* = 0.0134). There was also a significant increase in the level of phospholipids (treatment effect: *F*_1,14_ = 9.870; *p* = 0.0072), whereas the concentration of different classes of lipid metabolites, phosphatidylethanolamines (treatment effect: *F*_1,14_ = 7.904; *p* = 0.0139), lysophospholipids (treatment effect: *F*_1,14_ = 5.449; *p* = 0.0350), diacylglycerols (treatment effect: *F*_1,14_ = 6.320; *p* = 0.0248), and sphingomyelins (treatment effect: *F*_1,14_ = 5.684; *p* = 0.0318), was decreased.

### Effect of Long-Term Low-Dose Δ^9^-THC Treatment on CB1 Receptor Levels

To determine whether our treatment regimen altered CB1 protein levels, we compared CB1 protein expression between vehicle and THC-treated animals in the cortex, hippocampus, and adipose tissue of animals at days 14, 28, and 42. As shown in Figure S6, there was no significant difference in CB1 receptor expression between groups, suggesting that our long-term low-dose Δ^9^-THC treatment did not result in downregulation of CB1 receptor expression.

### Output of the Minipumps In Vitro

We found a highly significant difference in the effect of Δ^9^-THC treatment on the metabolome between day 14 and day 28; therefore, we wondered whether the output of the Alzet osmotic minipumps changed in this period. However, we found no significant change in the output of the minipumps measured weekly for 4 weeks (*F*_3,23_ = 1.792; *p* = 0.1769).

## Discussion

Our present results now suggest that Δ^9^-THC-induced consecutive bidirectional changes in mTOR signaling and in the metabolome may play a significant role in the positive effect of Δ^9^-THC treatment against brain aging. Here, we have shown that a low-dose long-term Δ^9^-THC treatment leads to a temporary increase in mTOR activity and mobilization of energy resources, thus triggering the formation of new synapses. This phase is followed by a reduced energy expenditure and reduced mTOR signaling in the adipose tissue, probably due to the depletion of resources in the first phase. Through this mechanism, Δ^9^-THC treatment combines the pro-cognitive effect of an mTOR activation with the antiaging effect of mTOR activity blockade. Our data now suggest that a long-term low-dose Δ^9^-THC treatment could be a particularly effective treatment strategy against brain aging. These results are in line with our previous studies showing that long-term low-dose Δ^9^-THC treatment increased the cognitive abilities of old mice by stimulating synapse dynamics and brain transcriptional activity through epigenetic mechanisms. Our study shows that changes in the metabolome and mTOR activity correlate with increased synapse densities. Further studies are needed to determine the extent to which this mechanism contributes to the antiaging effects of THC.

The effect of Δ^9^-THC treatment was strictly time- and tissue-dependent. Thus, mTOR activity in the brain was significantly higher at day 14 in Δ^9^-THC-treated mice than at day 28 despite the continuous treatment. In the adipose tissue, we found a lower level of mTOR phosphorylation in Δ^9^-THC-treated mice at all time points. The fact that Δ^9^-THC treatment reduced mTOR activity was unexpected because previous studies found a significant increase in mTOR activities in the brain of Δ^9^-THC-treated mice,^17^ which was responsible for the amnestic effect of high doses of Δ^9^-THC. Our study now shows that a long-term low-dose Δ^9^-THC treatment in aged mice has a bidirectional effect on mTOR activity in the brain: an initial increase followed by a downregulation. Interestingly, both changes can contribute to the positive effect of Δ^9^-THC on the aging brain. mTOR pathway activity contributes to synaptic plasticity and the formation of new synapses by facilitating translation and lipid biosynthesis.^15^ Thus, the initial increase in mTOR activity in Δ^9^-THC-treated animals can be responsible for the formation and stabilization of synapses, which we previously observed after long-term low-dose Δ^9^-THC-administration in old animals.^3,4^ A number of studies showed that a decreased mTOR activity, which was present at the late phase of the treatment period and after the cessation of the administration, has an antiaging effect by upregulating autophagy.^21,22^ Blocking mTOR activity is beneficial for the aging brain due to its antiaging effects, but it can also impair cognitive function by reducing synaptogenesis. Long-term low-dose THC treatment overcomes this problem by having a bidirectional effect on mTOR activity and metabolic processes.

Our results suggest that even a shorter, 14-day Δ^9^-THC treatment would probably be sufficient to induce a similar upregulation of mTOR activity, energetics, and synaptogenesis as the 28-day treatment in our present experiment. Thus, it is highly unlikely that Δ^9^-THC treatment for longer than 28 days would affect brain metabolomics differently than the 28-day treatment protocol. Nevertheless, it cannot be excluded that a longer treatment protocol would be even more beneficial for body aging parameters than the 28-day protocol. The longer treatment schedule was necessary to achieve a beneficial antiaging effect in the periphery, as a significant downregulation of mTOR activity in adipose tissue and an increase in blood plasma concentrations of antiaging metabolites were only present after 28 days of treatment. Importantly, CB1 receptor levels in our target areas did not change with the treatment regimen. Whether prolonged treatment with 3 mg/kg/day of Δ9-THC would result in a downregulation of CB1 receptor activity is an open question, although it is rather unlikely in the light of our present results (Figure S6).

We expected that the highly lipophilic Δ^9^-THC affects mTOR activity, besides in the brain, also in the adipose tissue, because it accumulates in the fat tissue^23^ and fat tissues express CB_1_ receptors at a high level.^24^ However, the direction and dynamics of the change were unexpected because we found decreased mTOR activity and reduced levels of amino acid and carbohydrate metabolites at the end of the treatment period. Lower mTOR phosphorylation is associated with energy mobilization, whereas previous studies clearly showed that activation of CB_1_ receptors leads to enhanced energy storage and reduced energy expenditure.^25^ CB_1_ receptor activity on adipocytes regulates important aspects of energy expenditure: Decreased activity is associated with lower energy storage,^26^ whereas increased activity favors energy accumulation.^27^ We hypothesize that increased mTOR activity in the brain mimicked energy abundance and led to an overall increase in the level of synthetic processes. The high energy and metabolite demand of exacerbated synthetic activities then depleted the energy resources, which, in turn, reduced mTOR activity.

Similar to the effect of mTOR signaling, the effect of Δ^9^-THC on the metabolome changed with time and varied between tissues: the peak of changes in the metabolome was on day 14 in the hippocampus, on day 28 in the blood plasma, and on day 42 in the fat tissue. The predominant change was an increase in metabolite levels in the hippocampus, whereas in the plasma, it was just the opposite as in the brain—a decline. In the adipose tissue, the direction of change was dependent on the metabolite family—increase in lipids and decline in carbohydrates and amino acids. Importantly, although the affected compound families differed between the tissues, we generally observed an increase in the concentration of compounds with known antiaging effect, compounds associated with histone acetylation (like α-ketoglutarate), and compounds involved in energy expenditure (most of the compounds involved in glycolysis and citric acid cycle).

One of the most prominent metabolic changes was the significantly enhanced carbohydrate metabolism and energy production in the hippocampus at day 14, as shown by the increased concentration of metabolites of glycolysis, fructose, pentose, and pentose phosphate metabolism, as well as the citric acid cycle. The fact that Δ^9^-THC treatment can enhance brain metabolism is not unprecedented because previous studies found a dose-dependent increase of brain glucose uptake and gluconeogenesis after Δ^9^-THC treatment in rodents^11,19^ as well as enhanced glucose metabolism,^20^ enhanced oxygen extraction rate, and an increased cerebral metabolic rate in the brain of human cannabis users.^12^

CB_1_ receptor activation can influence cell energetics on the cellular level through the regulation of mTOR activity,^17^ and on an organismal level through endocrine mechanisms.^28^ Further significant effects of long-term Δ^9^-THC treatment on the metabolome included an increase in several classes of lipid metabolites in the hippocampus and blood plasma at day 14, and at day 42 in the fat tissue. An interesting aspect of these changes was the increase of the endogenous ligand of the CB_1_ receptor, 2-arachidonoylglycerol (2-AG), and other endocannabinoids in the hippocampus at day 14 in Δ^9^-THC-treated mice, suggesting a further activation of the cannabinoid system by this pathway.^29^ Additionally, the levels of amino acids and their metabolites were significantly increased in the hippocampus. Thus, at the same time when the synaptic rearrangement took place after long-term low-dose Δ^9^-THC treatment,^3^ all of the prerequisites of the formation of new synapses—increased energy production, high-level amino acids and lipids for the production of proteins and membranes—were also present.

Δ^9^-THC treatment-induced metabolic changes also generate substances like long-chain and polyunsaturated fatty acids, compounds with a well-documented antiaging effect.^30^

It was previously shown that enhanced histone acetylation is a key for the long-term effects of Δ^9^-THC treatment, but the mechanism of this epigenetic effect was not clear. Our study now identified a group of metabolites with a potential effect on histone modifications whose level was changed after Δ^9^-THC treatment: Citric acid cycle intermediates like α-ketoglutarate, succinate, and fumarate regulate the level of histone methylation.^31^ It was suggested that α-ketoglutarate, a cofactor of histone demethylases (i.e., epigenetic enzymes), links the nutritional and epigenetic states and regulates a number of cellular antiaging homeostatic processes.^32^ Accordingly, α-ketoglutarate supplementation extended the life span and delayed age-related pathologies in several animal models of aging.^33^ These results suggest that metabolic changes after long-term Δ^9^-THC treatment may contribute to histone modifications, critical for the antiaging effect of Δ^9^-THC.

Our previous studies showed that long-term low-dose Δ^9^-THC treatment has an opposite effect on the brain of young and old animals: Δ^9^-THC-treated old mice showed an improved learning ability and enhanced synapse densities, whereas the same treatment slightly impaired the memory and destabilized spines in young animals.^3,4^ We now show that the effect of Δ^9^-THC on the metabolome was also strongly dependent on age: most of the compound classes influenced by Δ^9^-THC in old mice were also affected in young animals, but in the opposite direction! Thus, this age-dependent effect of Δ^9^-THC on the metabolome may contribute to the age-dependent effects of Δ^9^-THC. Previous studies showed that the endocannabinoid system activity in old mice is lower than that in young animals: the coupling^34^ and expression of CB_1_ receptors^35^ are substantially reduced in aged animals. Thus, we hypothesized that Δ^9^-THC treatment in old animals restores CB_1_ signaling to the physiological range, whereas the same treatment in young animals increased it above the normal range. Whether this effect is responsible for the observed striking difference in Δ^9^-THC effects between the age groups is not clear and requires further studies.

Despite the unaltered food intake and motor activity in Δ^9^-THC-treated animals, we detected several changes characteristic to physical activity, caloric restriction, or ketogenic diet, lifestyle strategies with antiaging effects in rodents^36^ and in humans.^37^

The reason why such diverse lifestyle factors exert similar antiaging effects may be their similar effect on the metabolome. It was hypothesized that altered activity of mTOR^38,39^ and epigenetic modifications^40,41^ connect these metabolic changes to the altered expression of genes involved in cellular homeostasis and defense.^42^ Long-term administration of low doses of Δ^9^-THC may thus support the positive effects of physical activity or a low-caloric diet in the elderly, an age group where health issues can impede the full application of these strategies.

## Materials and Methods

### Animals and Experimental Groups

Male C57BL6/J 4- and 18-month-old mice (Janvier) were used for the experiments. The mice were randomly selected for the treatment groups. The animals used for the metabolome analysis or validation were treated with vehicle or 3 mg/kg/day Δ^9^-THC through subdermal osmotic minipumps (*n* = 8 per group). To test the relation between Δ^9^-THC dose and plasma N-acetylated amino acid concentration, a separate group of 18-month-old male C57BL6/J mice (*n* = 12 per group) was used. All experiments were approved by the North Rhine-Westphalia State Environment Agency (LANUV, Landesamt fuer Natur, Umwelt and Verbraucherschutz, license nr: AZ: 84-02.04.2017.A190) and were performed in accordance with the relevant guidelines and regulations.

### Experimental Design

Osmotic minipumps (Alzet, CA) filled with Δ^9^-THC or vehicle (ethanol:cremophor:saline in the volume ration of 4:4:2) were implanted subcutaneously 2 weeks after arrival of the animals to the animal facility at day 0. Their body weight and food consumption were registered every third day during the experiment starting at day 4 and they were kept single-housed from this time point. Additionally, home cage activity was monitored through the Inframot system (TSE-Systems, Germany) for 4 days in 10-10 randomly selected mice during treatment (day 21) and withdrawal (day 35) phases of the experiment. To test the effect of Δ^9^-THC treatment on the metabolome 18-month-old mice were killed with decapitation on days 14, 28, and 42 (at the 14th day of the withdrawal phase, the pumps run a maximum of 28 days), whereas all of the 4-month-old mice were sacrificed at day 28. Blood plasma, visceral white fat tissue, cortex, and hippocampus were isolated, frozen, and maintained at −80 °C until processed. Blood plasma, visceral white fat tissue, and hippocampus were used for the metabolome analysis, and cortex was used to control the level of mTOR phosphorylation and the amount of synaptic proteins using Western blotting. To test whether there is a similar change in mTOR activity or synaptic protein levels in the cortex and hippocampus, we treated a separate cohort of 14–14 mice as described above. The animals were killed on day 14, and their hippocampi were prepared for automated Western blotting.

For the analysis of dose effects on plasma N-acetylated amino acid levels, an additional group of 18-month-old male C57BL6/J mice were treated with vehicle or Δ^9^-THC at doses of 0.3, 1, and 3 mg/kg/day using osmotic minipumps as described above. On day 28, the animals were decapitated, and blood plasma was collected and kept at −80 °C until further analysis.

### Statistical Analyses

For the analysis of time-dependent changes in the metabolome, following normalization to volume extracted (plasma), log transformation, and imputation of missing values, if any, with the minimum observed value for each compound, a two-way ANOVA and Welch’s two-sample *t* tests were used to identify biochemicals that differed significantly between vehicle and Δ^9^-THC-treated groups. Additionally, to identify the biochemical pathways and molecules affected by Δ^9^-THC treatment, two-way repeated ANOVA was used (between factor: treatment, within factor: molecule) separately to time points and biochemical pathways. To analyze age effect, two-way ANOVA was used (main factors: treatment and time for mTOR and age and molecule for the age effect). Lastly, to analyze the effect of Δ^9^-THC treatment on mTOR phosphorylation, on the levels of synaptophysin, PSD-95, or CB1 receptors, Student’s unpaired *t* test was used.

### Analysis of Synaptic Protein Levels and mTOR Phosphorylation Using Automated Western Blotting (WES)

Deep frozen tissue was homogenized using a CryoMill device (Retsch, Germany), and the frozen powder was kept at −80 °C until further processing. On the test day, cortex or hippocampus samples were homogenized in ice-cold radioimmunoprecipitation assay (RIPA) buffer (approximately 100 mg tissue/mL) containing a cocktail of protease inhibitors (cOmplete, EDTA-free, Roche Diagnostics GmbH, Germany) and phosphatase inhibitors (Pierce Phosphatase Inhibitor, Thermo Schientific, IL) and used directly for WES. Fat tissue samples were homogenized in ice-cold RIPA buffer without Triton X-100, but containing protease and phosphatase inhibitors as above. Samples were centrifuged at 6000*g* for 15 min at 4 °C. The fat layer was removed from the top of the sample, and Triton X-100 was added at a final concentration of 1% (v/v) to the remaining aqueous layer. The sample was mixed and kept on ice for 60 min. After incubation, the sample was subsequently centrifuged at 12,000*g* for 15 min at 4 °C. Samples were used for WES analysis after removal of the upper lipid layer.

The total protein concentration was measured by using a bicinchoninic acid (BCA) protein assay kit (Thermo Scientific, Lausanne, Switzerland). 0.03–0.32 mg/mL protein was used for WES analysis using 12–230 kDa cartridge kits for the synaptic proteins and 66–440 kDa cartridge kits for mTOR and phosphor-mTOR (ProteinSimple WES, Germany). The primary antibodies for the synaptic proteins PSD-95 (1:500, ab18258, Abcam, The Netherlands), synaptophysin (1:25, ab32127, Abcam, The Netherlands), mTOR1 (1:200, 2983, Cell Signaling Technology, The Netherlands), and phospho-mTOR1 (1:25 for fat and 1:100 for cortex or hippocampus, 5536, Cell Signaling Technology, The Netherlands) were used. Anti β-actin (1:500 for PSD-95 and 1:200 for synaptophysin, NB600-532, Novus Biologicals, Centennial, CO) served as an internal control for the synaptic proteins. Samples were analyzed using Compass software (ProteinSimple, version 4.0.0, Wiesbaden, Germany). Electropherogram and virtual blots were checked and evaluated for each sample. A chemiluminescent signal was quantified by the software, and the area of the samples was normalized to β-actin for the synaptic proteins and to mTOR for phospho-mTOR.

### Analysis of CB1 Receptor Protein Levels Using Western Blotting

Frozen hippocampus, cortex, and adipose tissue samples of vehicle and THC-treated mice on days 14, 28, and 42 were lysed in 1% SDS buffer (Sigma-Aldrich, Munich, Germany) containing protease inhibitor (Complete Mini, Roche), sonicated, and clarified by centrifugation (13,000 rpm for 10 min). Protein concentrations were determined using a BCA Protein Assay Kit (Pierce). Equal amounts of protein were run on NuPAGE Bis-Tris 4–12% gradient gels (Invitrogen, Carlsbad, CA). In preliminary experiments, we determined the range where the relation between the protein concentration and signal intensity is linear for each antibody. The proteins were subsequently blotted onto PVDF membranes using an iBlot Dry Blotting System (Invitrogen, Carlsbad, CA). The blots were incubated with primary antibodies to CB1 (101500; 1:1000; Cayman), β-actin (1:10,000; aA5441; Sigma), or GAPDH (1:2000; ab9484; Abcam). The blots were then incubated with peroxidase-conjugated secondary antibodies followed by the ECL substrate (Pierce). Images were created using the ChemiDoc Imaging System (Bio-Rad Laboratories), and the quantification was performed using the ImageLab software (Bio-Rad Laboratories). Signal intensities from the cortex and hippocampus were normalized to GAPDH, from adipose tissue to β-actin.

### Analysis of the Metabolome

The metabolome analysis was performed by Metabolon, Inc. (NC). For that, samples were prepared using the automated MicroLab STAR system from the Hamilton Company. Several recovery standards were added prior to the first step in the extraction process for quality control purposes. To remove protein, to dissociate small molecules bound to proteins or trapped in the precipitated protein matrix, and to recover chemically diverse metabolites, proteins were precipitated with methanol under vigorous shaking for 2 min (Glen Mills GenoGrinder 2000) followed by centrifugation. The resulting extract was divided into five fractions: two for analysis by two separate reverse phase (RP)/UPLC-MS/MS methods with positive-ion mode electrospray ionization (ESI), one for analysis by RP/UPLC-MS/MS with negative-ion mode ESI, one for analysis by HILIC/UPLC-MS/MS with negative-ion mode ESI, and one sample was reserved for backup. Samples were placed briefly on a TurboVap (Zymark) to remove the organic solvent. Sample extracts were stored overnight under nitrogen before preparation for analysis with ultrahigh-performance liquid chromatography-tandem mass spectroscopy (UPLC-MS/MS): All methods utilized a Waters ACQUITY ultraperformance liquid chromatography (UPLC) and a Thermo Scientific Q-Exactive high resolution/accurate mass spectrometer interfaced with a heated electrospray ionization (HESI-II) source and an Orbitrap mass analyzer operated at 35,000 mass resolution. The sample extract was dried and then reconstituted in solvents compatible with each of the four methods. Each reconstitution solvent contained a series of standards at fixed concentrations to ensure injection and chromatographic consistency. One aliquot was analyzed using acidic conditions and a positive-ion mode and chromatographically optimized for more hydrophilic compounds. In this method, the extract was gradient-eluted from a C18 column (Waters UPLC BEH C18–2.1 mm × 100 mm, 1.7 μm) using water and methanol, containing 0.05% perfluoropentanoic acid (PFPA) and 0.1% formic acid (FA). Another aliquot was also analyzed using acidic positive-ion mode conditions; however, it was chromatographically optimized for more hydrophobic compounds. In this method, the extract was gradient-eluted from the same aforementioned C18 column using methanol, acetonitrile, water, 0.05% PFPA, and 0.01% FA and was operated at an overall higher organic solvent content. An additional aliquot was analyzed using optimized basic negative-ion mode conditions with a separate dedicated C18 column. The basic extracts were gradient-eluted from the column using methanol and water, however, with 6.5 mM ammonium bicarbonate at pH 8. The fourth aliquot was analyzed via negative ionization following elution from a HILIC column (Waters UPLC BEH Amide 2.1 mm × 150 mm, 1.7 μm) using a gradient consisting of water and acetonitrile with 10 mM ammonium formate, pH 10.8. The MS analysis alternated between MS and data-dependent MSn scans using dynamic exclusion. The scan range varied between methods but covered 70–1000 *m*/*z*.

Raw data were extracted, peak-identified, and quality control processed using Metabolon’s hardware and software. Compounds were identified by comparison to library entries of purified standards or recurrent unknown entities. Library matches for each compound were checked for each sample and corrected if necessary. Peaks were quantified using an area-under-the-curve calculation. For studies spanning multiple days, a data normalization step was performed to correct variation resulting from instrument interday tuning differences. Essentially, each compound was corrected in run-day blocks by registering the medians to equal one (1.00) and normalizing each data point proportionately.

### Measurement of Osmotic Minipump Output In Vitro

Osmotic minipumps were filled with THC as in the in vivo experiment (3.78 mg of THC in 1 mL of ethanol:cremophor:saline 4:4:2 mixture) and placed individually in Falcon tubes containing 2 mL of saline. The tubes containing the minipumps in saline were kept at 37 °C for 28 days. The liquid containing the saline and the released THC solution was removed every 7 days and frozen until further analysis. The tubes were refilled again with 2 mL of saline, and this process was repeated 4 times, until day 28.

### Analysis of Δ^9^-THC Levels in the In Vitro Experiment

(−)-Δ ^9^-THC and (−)-Δ^9^-THC-d3 were obtained from Cerilliant (Round Rock, TX). Acetonitrile (hypergrade for LC-MS) and ammonium acetate were purchased from Merck (Darmstadt, Germany). Acetic acid was purchased from Sigma-Aldrich (Steinheim, Germany), and methanol (for high-performance liquid chromatography (HPLC), gradient grade) was obtained from Honeywell Riedel-de-Han (Seelze, Germany).

Quantification of Δ^9^-THC was performed by using high-performance liquid chromatography (HPLC) coupled to triple quadrupole mass spectrometry (LC-MS/MS). The LC-MS/MS apparatus consisted of an LC-20 series HPLC system (binary pump, degasser, column oven, and autosampler; Shimadzu, Duisburg, Germany) coupled to an API 4000 QTrap mass spectrometer (Sciex, Darmstadt, Germany). The analysis was performed using negative mode ESI and multiple reaction monitoring (MRM). The following settings were utilized: collision gas, nitrogen; collision gas, high; curtain gas, 25 psi; ion source gas 1, 40 psi; ion source gas 2, 60 psi; ion spray voltage: −4500 V; and temperature, 500 °C. Two ion transitions each were used for the detection and identification of Δ^9^-THC and Δ^9^-THC-d3 (Δ9- THC: 313.2–245.0 (target), 313.2–191.1 (qualifier); Δ^9^-THC-d3: 316.2–248.1 (target), 316.2–194.1 (qualifier)).

Chromatographic separation was performed using a NUCLEODUR C18 Isis (5 μm, 4.6 mm × 150 mm) column (Macherey-Nagel, Düren, Germany) with gradient elution (total flow, 0.8 mL/min). Eluents A and B consisted of 5 mM ammonium acetate in deionized water (adjusted to pH 5.7 with acetic acid) and acetonitrile/methanol (1:9, v/v, adjusted to pH 5.7 with acetic acid), respectively. The following gradient program was used: starting at 75% B, keeping it for 2 min, linear gradient to 90% B within 6 min, keeping it for 5 min, and back to 75% B within 1 min, followed by equilibration for 2 min. The injection volume was 30 μL. The column was maintained at 40 °C.

The in vitro sample solutions were initially diluted with methanol (1:99, v/v). A 50 μL aliquot of the respective methanolic sample dilution was mixed with 50 μL of an internal standard solution containing 200 ng/mL Δ^9^-THC-d3. This mixture was immediately used for analysis.

A linear calibration (range of 5–1000 ng/mL) was established by analyzing appropriately concentrated solutions of Δ^9^-THC in methanol (50 μL of Δ^9^-THC solution mixed with 50 μL of the internal standard solution). Furthermore, two quality control samples (with Δ^9^-THC concentrations of 100 and 250 ng/mL) and a blank sample (consisting of 50 μL of methanol as well as 50 μL of the internal standard solution) were included in the analysis batch. Δ^9^-THC concentrations were quantified using peak area ratios (Δ^9^-THC/Δ^9^-THC-d3).

### Analysis of Plasma N-Acetylated Amino Acid Levels

Acetonitrile (cooled at 4 °C) containing 0.2% of formic acid (100 μL) was added to 100 μL of plasma sample. After centrifugation, the supernatant was transferred to HPLC vials and mass spectra were recorded on a QTrap 6500+ instrument (Sciex, Darmstadt) with an ESI-source coupled with an HPLC 1290 Infinity instrument (Agilent, Waldbronn) using a Synergy 4 μM HydroRP 150 × 2 column (Phenomenex). The column temperature was 30 °C. Sample solution (5 μL) was injected at a flow rate of 0.6 mL/min. The HPLC run started using 100% water (containing 0.1% formic acid) as the eluent for 1.5 min. Then, a gradient was applied, reaching 100% acetonitrile (containing 0.1% formic acid) within 2.5 min. The column was flushed for a further 2 min with 100% acetonitrile containing 0.1% formic acid, followed by 4 min of equilibration. Calibration curves ranging from 0 to 500 nM were recorded for each of the analytes, all of which showed a high linearity over the entire concentration range.

## Supporting Information Available

Long-term low-dose THC treatment did not influence body weight change and only slightly reduced food intake, significantly at day 28 and no change in the home cage activity or in the diurnal cycle at the end of the THC treatment or at the end of the withdrawal phase (Figure S1); similar effect of THC treatment on polyunsaturated fatty acid (PUFA) metabolite levels in the blood plasma of young (4-month-old) and old (18-month-old) animals at day 28 (Figure S2); dominant opposite change in branched-chain amino acid and lysine metabolite levels in the blood plasma of THC-treated young (4-month-old) and old (18-month-old) animals at day 28 (Figure S3); compound-specific opposite effect of THC treatment between young (4-month-old) and old (18-month-old) animals on the methionine and phenylalanine metabolite levels in the blood plasma at day 28 (Figure S4); compound-specific opposite effect of THC treatment between young (4-month-old) and old (18-month-old) animals on the tryptophan and branched-chain dipeptide metabolite levels in the blood plasma at day 28 (Figure S5); long-term low-dose THC treatment did not significantly alter CB1 receptor levels in the cortex, hippocampus, or adipose tissue (Figure S6); metabolite groups with significantly increased hippocampal levels after 14 days THC treatment according to two-way ANOVA (Table S1); metabolite groups with significantly changed levels in the blood plasma after 14 days THC treatment according to two-way ANOVA (Table S2); metabolite groups with significantly changed levels in the blood plasma after 28 days THC treatment according to two-way ANOVA (Table S3); and metabolite groups with significantly changed levels in blood plasma on day 42, after 14 days of THC withdrawal, according to two-way ANOVA (Table S4) (PDF)
