# Supplementary material for: Bidirectional Effect of Long-Term Δ9-Tetrahydrocannabinol Treatment on mTOR Activity and Metabolome
Source: ACS Pharmacol Transl Sci. 2024 Aug 14;7(9):2637–49. doi: 10.1021/acsptsci.4c00002 (PMC11406684; doi:10.1021/acsptsci.4c00002)
Supplement: Supplementary file 1 — pt4c00002_si_001.pdf [file pt4c00002_si_001.pdf]

# Supporting Information

**Bidirectional effect of long-term Δ⁹-tetrahydrocannabinol-treatment on mTOR activity and metabolome**

Andras Bilkei-Gorzo[1*], Britta Schurmann[1], Marion Schneider[2], Michael Kraemer[3], Prakash Nidadavolu[1], Eva C. Beins[1], Christa E. Müller[2], Mona Dvir-Ginzberg[4], Andreas Zimmer[1]

1. Institute of Molecular Psychiatry, Medical Faculty, University of Bonn, 53125 Bonn, Germany

2 Pharmaceutical Institute, University of Bonn, Bonn, Germany

3 Institute of Forensic Medicine, Medical Faculty, University of Bonn, Bonn, Germany

4 Institute of BioMedical and Oral Research, Faculty of Dental Medicine, Hebrew University of Jerusalem, Jerusalem, Israel.

* Correspondence: abilkei@uni-bonn.de

Table of Contents

Title page……………………….S 1
Figure S1……………………… S 2
Figure S2……………………… S 3
Figure S3……………………… S 4
Figure S4……………………… S 5
Figure S5……………………… S 6
Figure S6……………………… S 7
Table S1………………………….S 8
Table S2………………………….S12
Table S3………………………….S16
Table S4………………………….S20

# Figure S1

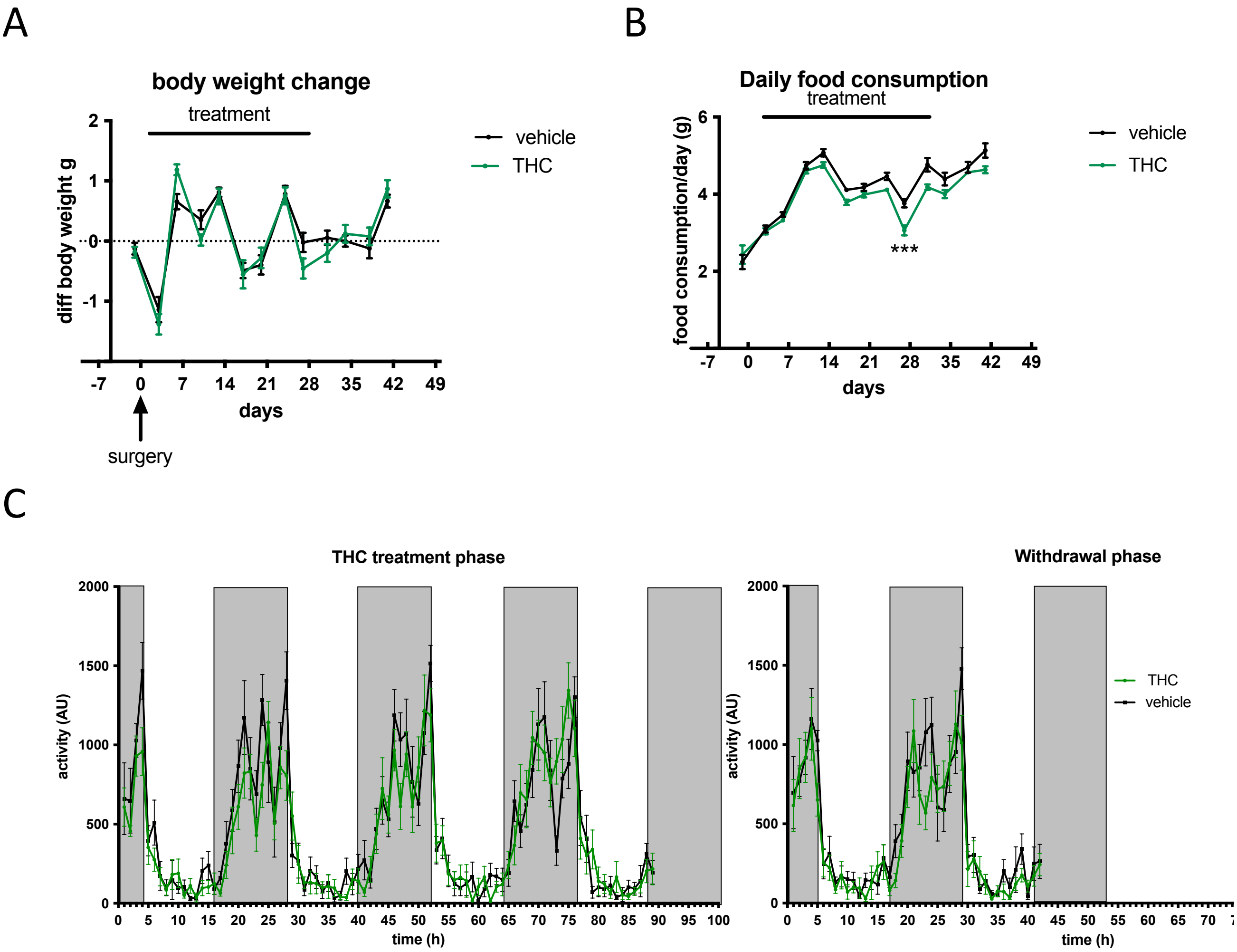


Long-term, low dose THC-treatment did not influence **(A)** body weight change and **(B)** only slightly reduced food intake – significantly at day 28. (**C**) No change in the home cage activity or in the diurnal cycle at the end of the THC-treatment or at the end of the withdrawal phase. Dots represent mean values, whiskers standard error of mean for panels A-C. *** $p < 0.001$ according to two-way ANOVA followed by Bonferroni t-test for panel B.

Figure S2

A

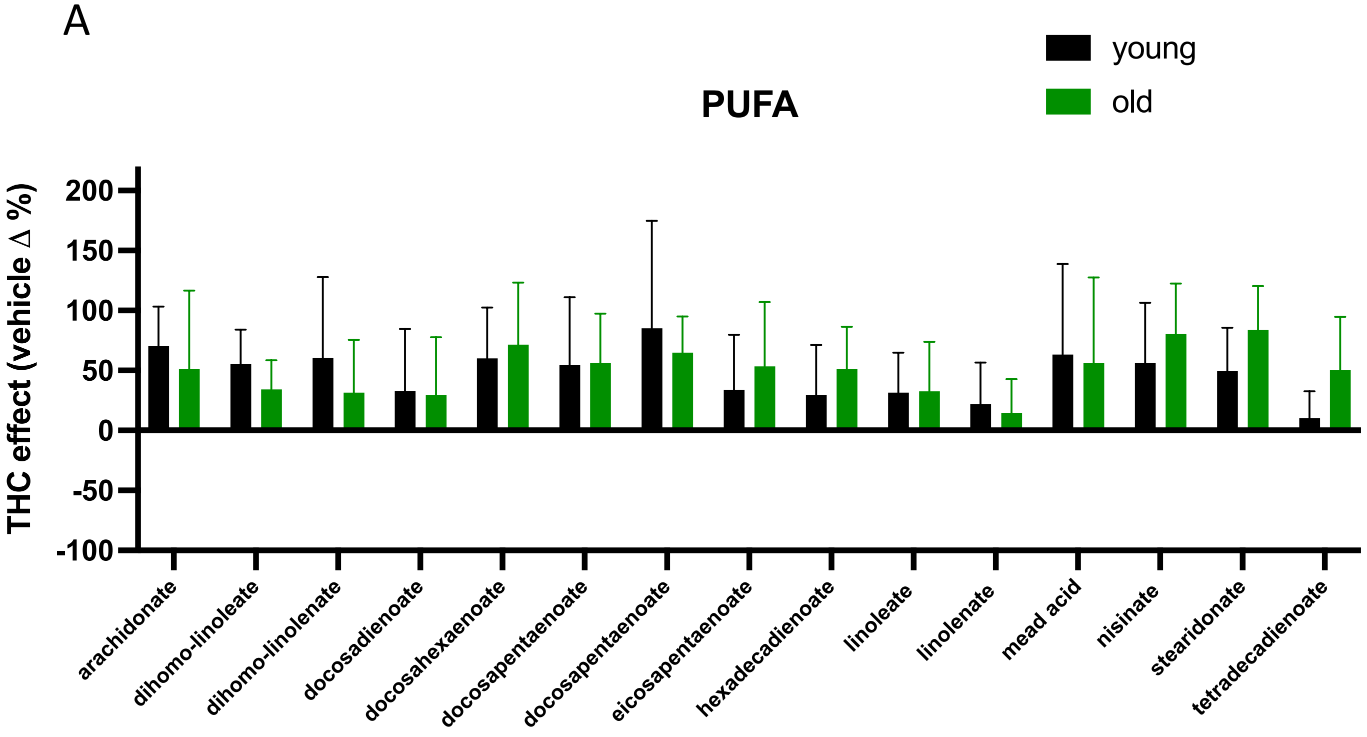


B

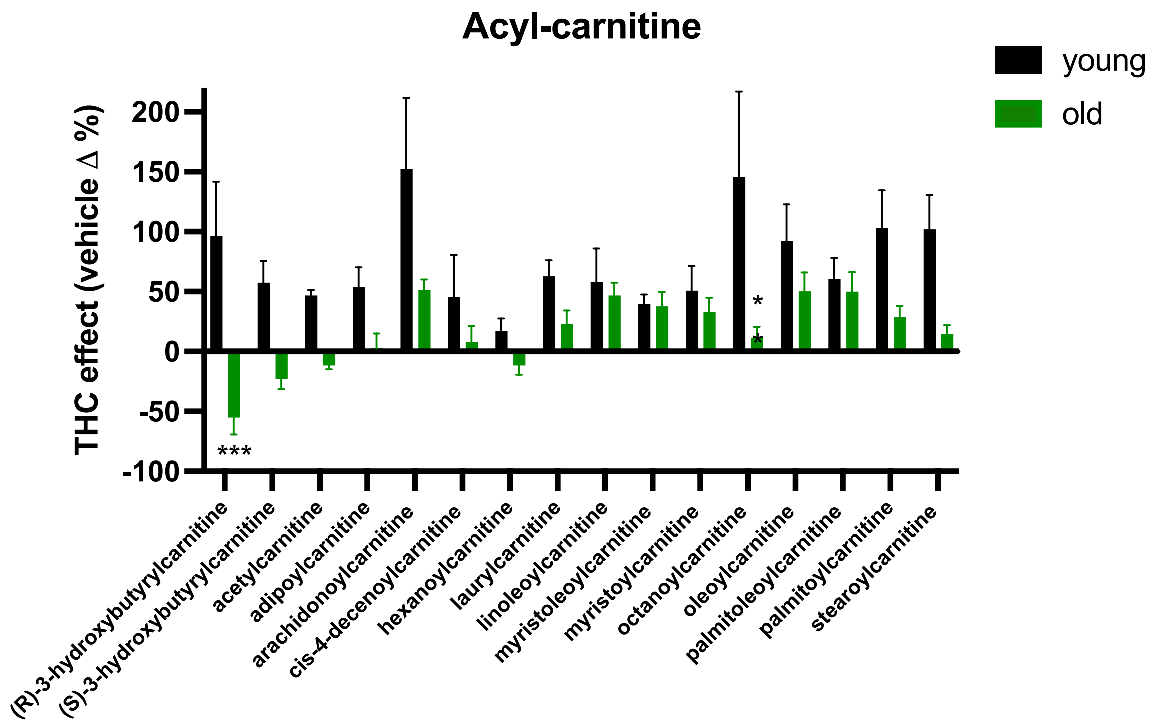


Similar effect of THC-treatment on **(A)** polyunsaturated fatty acid (PUFA) metabolite levels in the blood plasma of young (4-month-old) and old (18-month-old) animals at day 28. **(B)** Generally higher blood plasma levels of acyl-carnitine metabolites in THC-treated young than in old mice with the same treatment at day 28. Columns represent mean values, whiskers standard error. ** p < 0.01; *** p < 0.001 significant difference between the age-groups according two-way ANOVA followed by Bonferroni t-test.

Figure S3

A

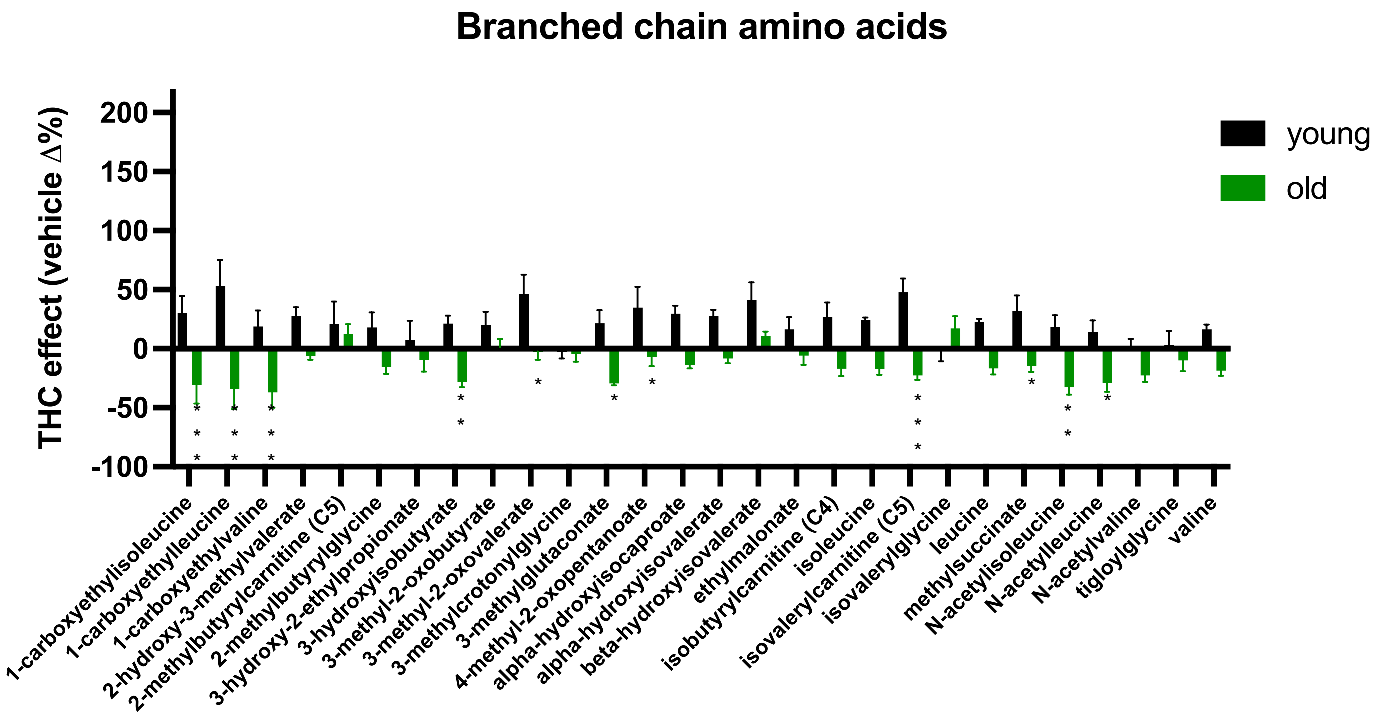


B

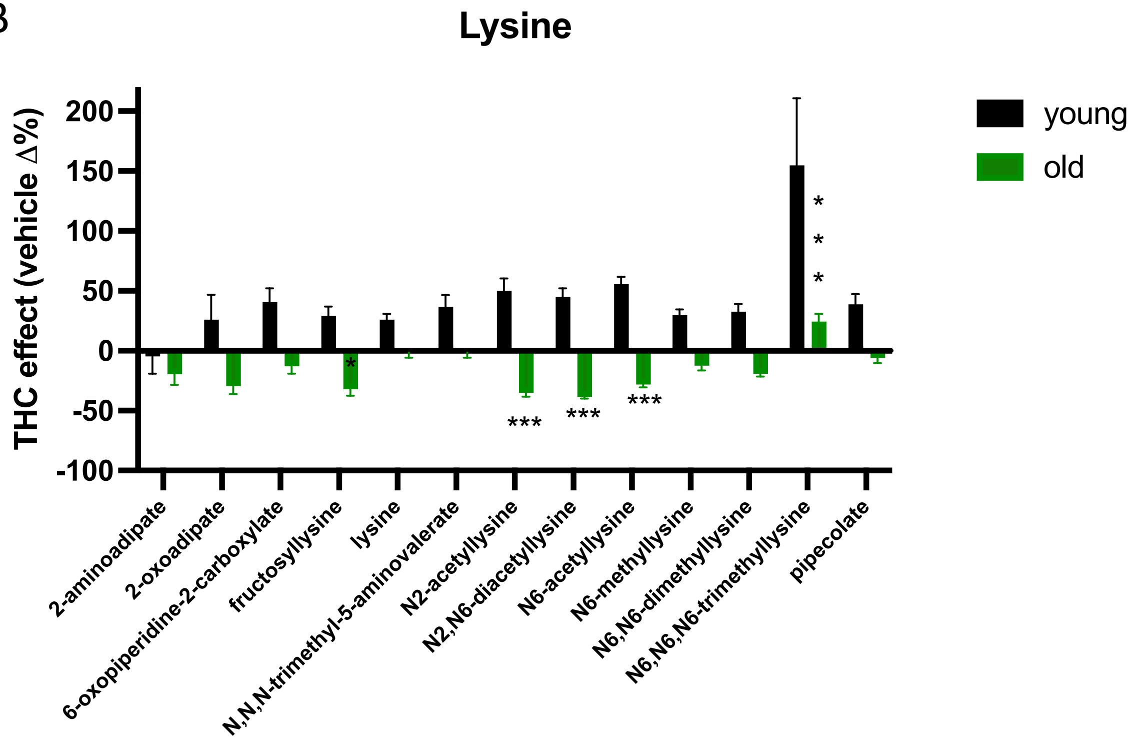


Dominant opposite change in **(A)** branched chain amino acid and **(B)** lysine metabolite levels in the blood plasma of THC-treated young (4-month-old) and old (18-month-old) animals at day 28. Columns represent mean values, whiskers standard error. * $p < 0.05$; ** $p < 0.01$; *** $p < 0.001$ significant difference in the effect of THC on the metabolite levels between young (4-month-old) and old (18-month-old) animals according two-way ANOVA followed by Bonferroni t-test.

Figure S4

A

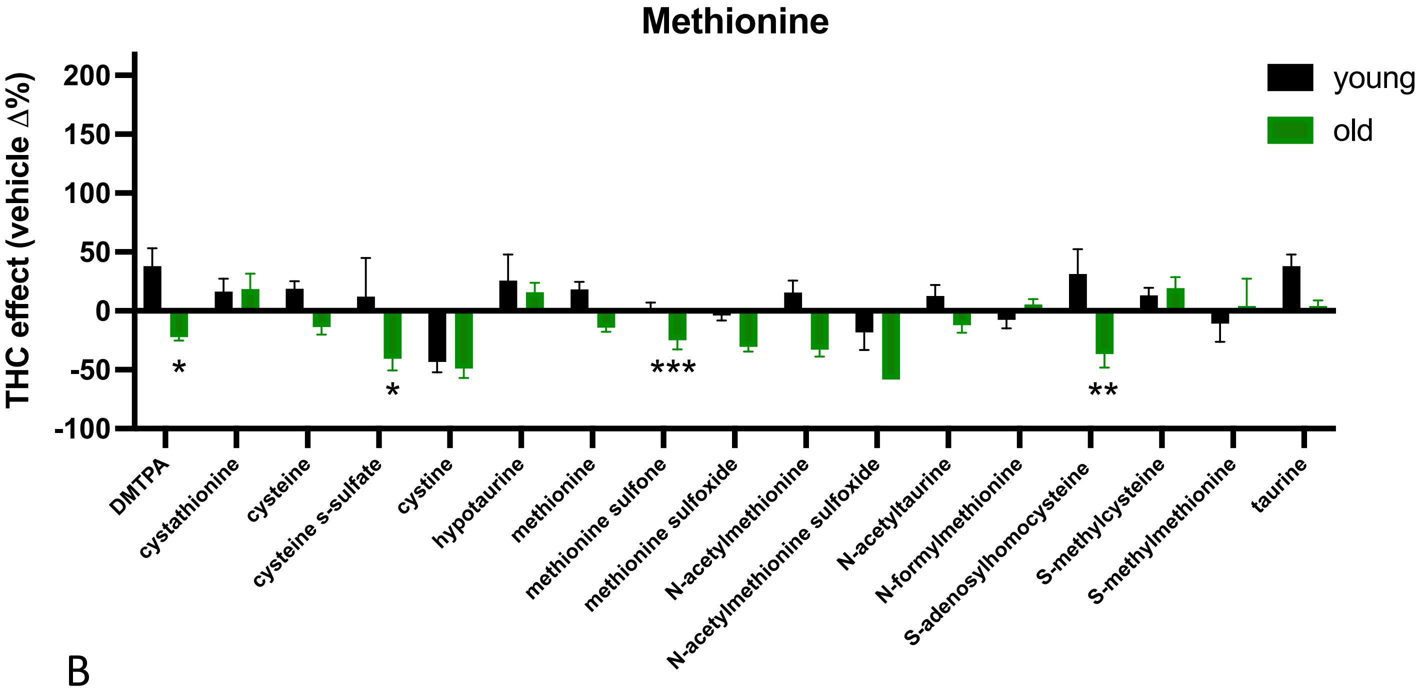


B

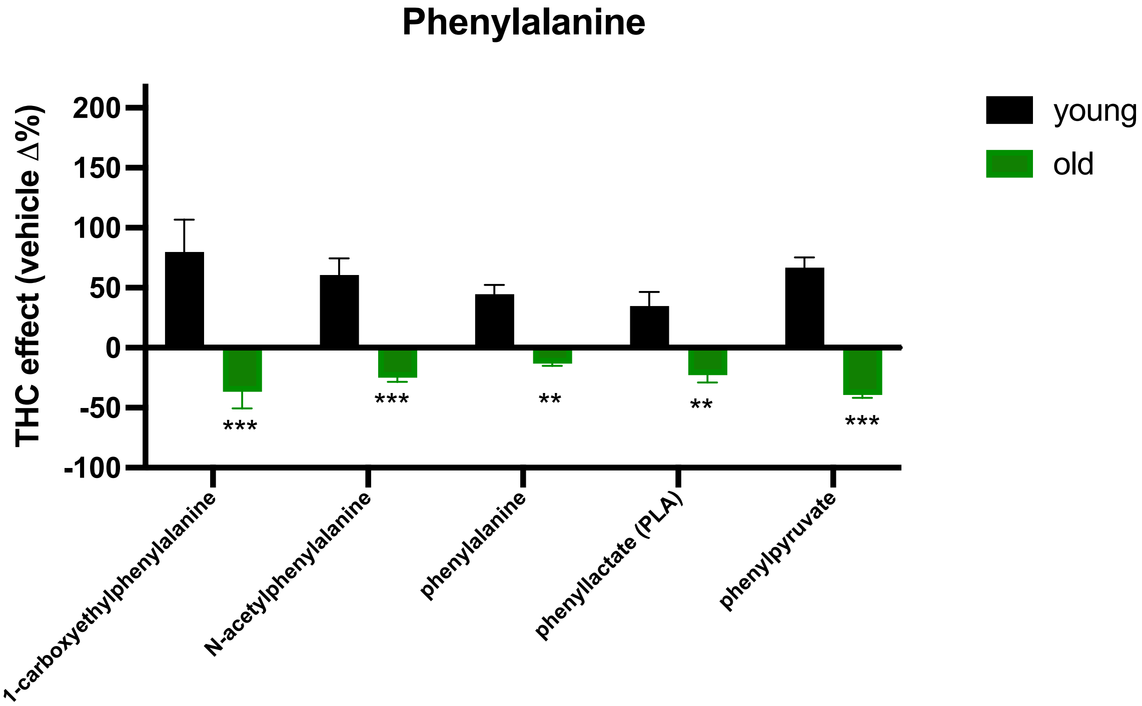


Compound-specific opposite effect of THC-treatment between young (4-month-old) and old (18-month-old) animals on the **(A)** methionine and **(B)** phenylalanine metabolite levels in the blood plasma at day 28. Columns represent mean values, whiskers standard error. * $p < 0.05$; ** $p < 0.01$; *** $p < 0.001$ significant difference in the effect of THC on the metabolite levels between young (4-month-old) and old (18-month-old) animals according two-way ANOVA followed by Bonferroni t-test.

Figure S5

A

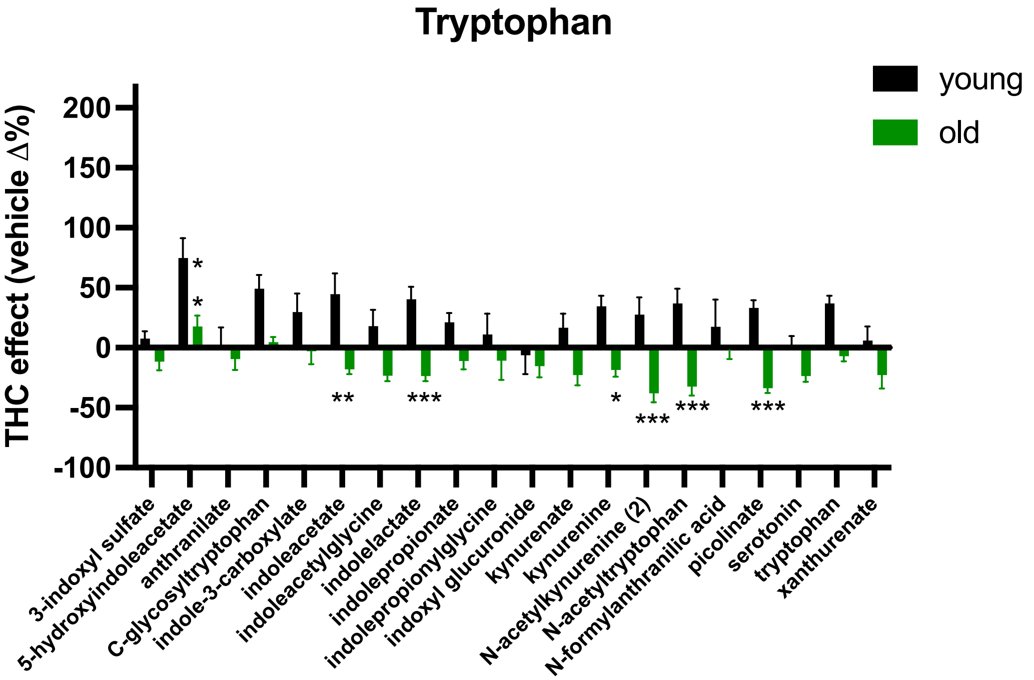


B

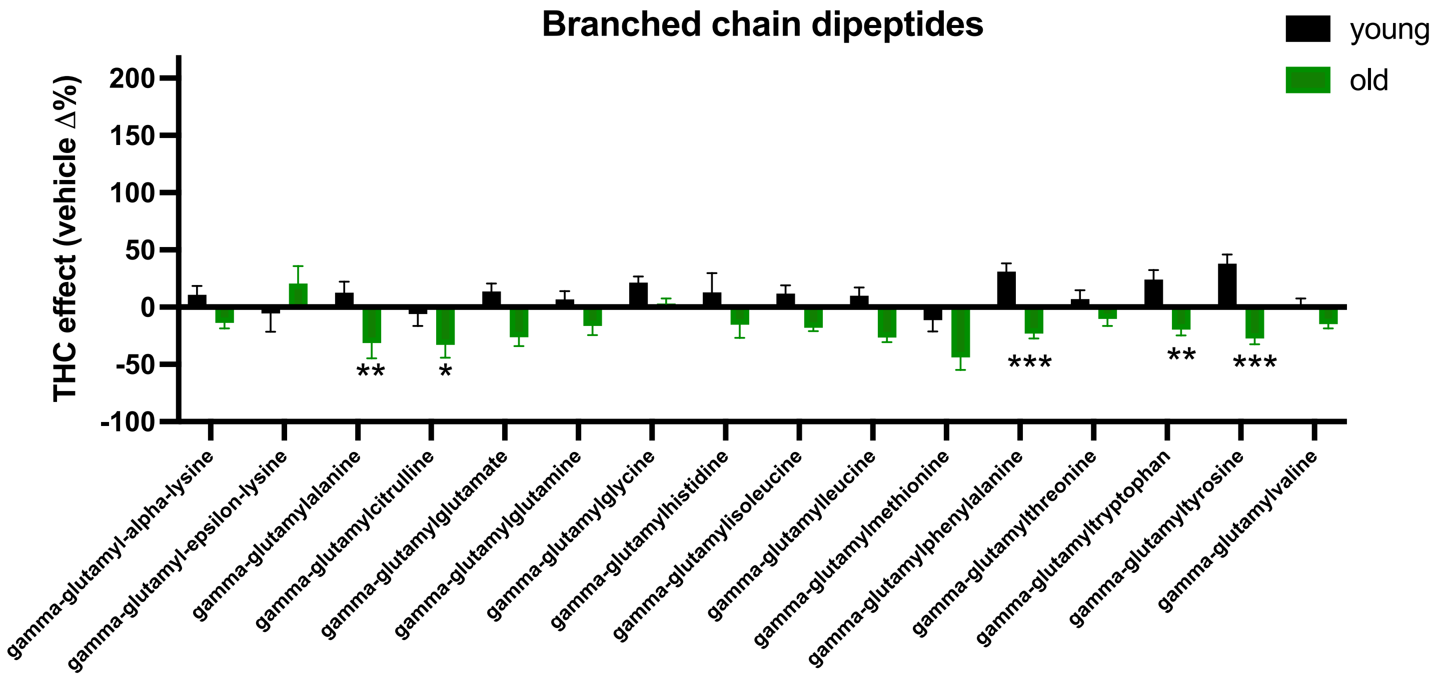


Compound-specific opposite effect of THC-treatment between young (4-month-old) and old (18-month-old) animals on the **(A)** tryptophan and **(B)** branched chain dipeptide metabolite levels in the blood plasma at day 28. Columns represent mean values, whiskers standard error. * $p < 0.05$; ** $p < 0.01$; *** $p < 0.001$ significant difference in the effect of THC on the metabolite levels between young (4-month-old) and old (18-month-old) animals according two-way ANOVA followed by Bonferroni t-test.

Figure S6

A

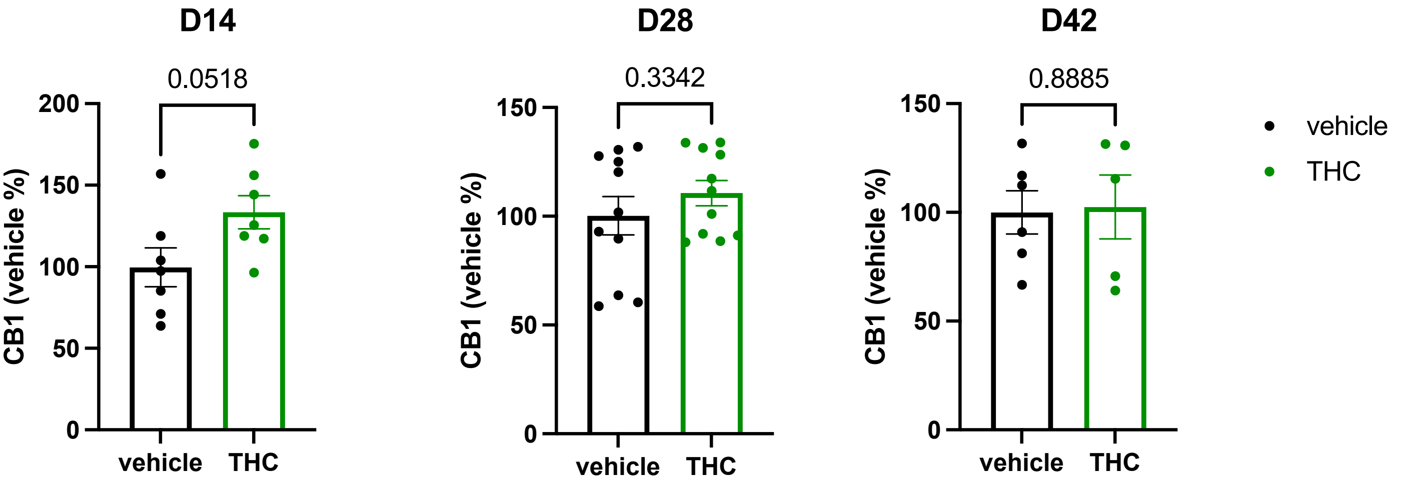


B

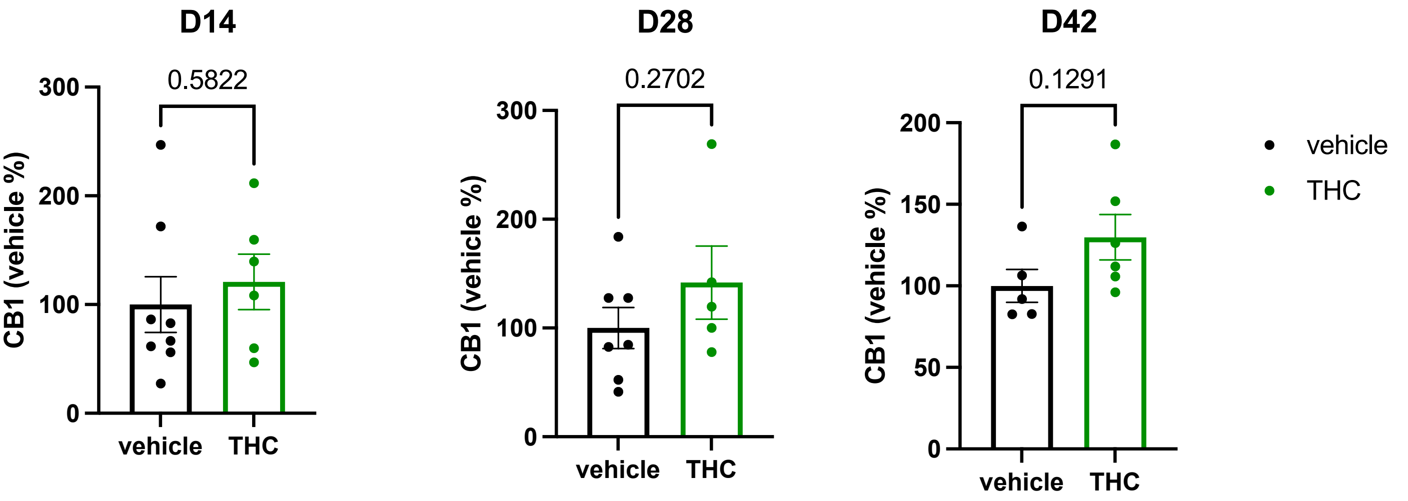


C

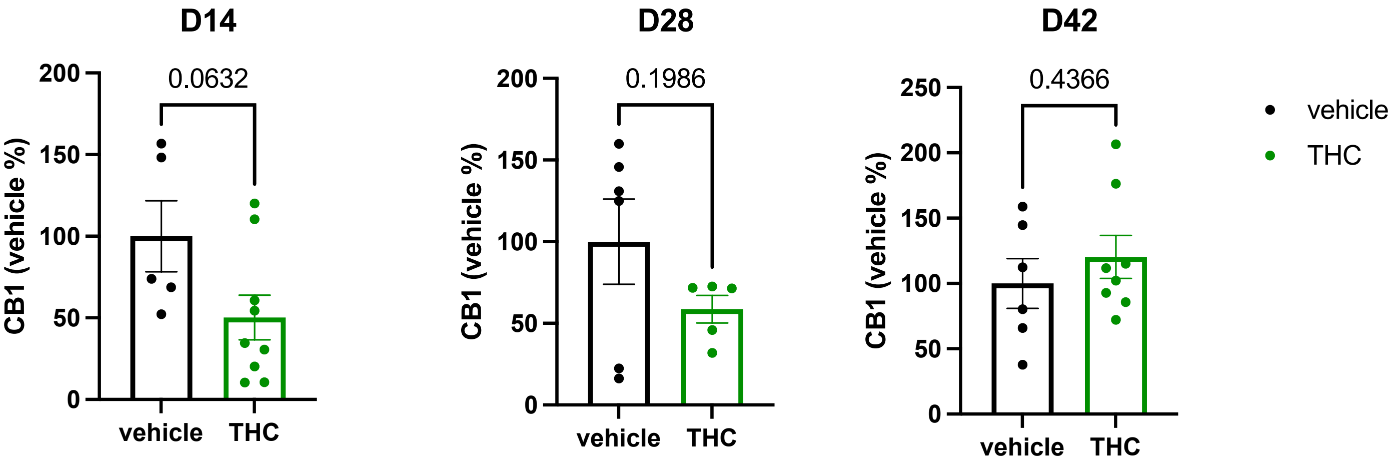


Long-term, low dose THC-treatment or withdrawal did not alter CB1 receptor levels in the **(A)** cortex, **(B)** hippocampus or (**C**) adipose tissue

S7

Table S1

## Pentose

| compound | THC (vehicle %) | | q value | individual p value |
|---|---|---|---|---|
| | mean | sd | | |
| arabinose | 116.683 | 30.824 | 0.218 | 0.242 |
| arabitol/xylitol | 105.424 | 37.818 | 0.711 | 0.902 |
| arabonate/xylonate | 138.008 | 59.858 | 0.042 | 0.020 |
| ribitol | 119.370 | 38.373 | 0.218 | 0.212 |
| ribonate | 129.878 | 35.904 | 0.074 | 0.047 |
| ribose | 125.313 | 29.665 | 0.076 | 0.060 |
| ribulonate/xylulonate | 140.477 | 50.904 | 0.022 | 0.007 |
| sedoheptulose | 142.963 | 24.924 | 0.015 | 0.002 |

## Fructose

| compound | THC (vehicle %) | | q value | individual p value |
|---|---|---|---|---|
| | mean | sd | | |
| fructose | 151.870 | 57.296 | 0.070 | 0.045 |
| galactonate | 124.575 | 18.688 | 0.070 | 0.038 |
| galactose 1-phosphate | 159.873 | 110.498 | 0.247 | 0.235 |
| mannitol/sorbitol | 110.505 | 8.731 | 0.070 | 0.033 |
| mannose | 164.415 | 75.537 | 0.070 | 0.037 |
| mannose-6-phosphate | 160.771 | 76.315 | 0.078 | 0.062 |

## Monoacylglycerol

| compound | THC (vehicle %) | | q value | individual p value |
|---|---|---|---|---|
| | mean | sd | | |
| 1-arachidonylglycerol (20:4) | 191.168 | 109.082 | 0.102 | 0.030 |
| 1-dihomo-linolenylglycerol (20:3) | 132.854 | 51.828 | 0.220 | 0.175 |
| 1-docosahexaenoylglycerol (22:6) | 170.008 | 90.594 | 0.102 | 0.048 |
| 1-oleoylglycerol (18:1) | 138.502 | 39.132 | 0.126 | 0.080 |
| 2-arachidonoylglycerol (20:4) | 185.673 | 98.728 | 0.102 | 0.042 |
| 2-oleoylglycerol (18:1) | 124.137 | 60.576 | 0.357 | 0.340 |

Metabolite groups with significantly increased hippocampal levels after 14 days THC-treatment according to two-way ANOVA. Individual metabolites with significantly altered concentration according to Welch´s t-tests are highlighted with red.

Table S1

## Lysophospholipids

| compound | THC (vehicle %) | | q value | individual p value |
|---|---|---|---|---|
| | mean | sd | | |
| 1-arachidonoyl-GPC (20:4n6) | 112.373 | 40.171 | 0.682 | 0.498 |
| 1-arachidonoyl-GPE (20:4n6) | 108.926 | 36.044 | 0.699 | 0.608 |
| 1-arachidonoyl-GPI (20:4) | 98.266 | 20.107 | >0.9999 | 0.953 |
| 1-linoleoyl-GPC (18:2) | 161.960 | 93.538 | 0.444 | 0.257 |
| 1-linoleoyl-GPG (18:2) | 126.183 | 45.487 | 0.336 | 0.120 |
| 1-oleoyl-GPC (18:1) | 114.888 | 35.603 | 0.455 | 0.283 |
| 1-oleoyl-GPE (18:1) | 104.426 | 32.093 | 0.891 | 0.811 |
| 1-oleoyl-GPG (18:1) | 140.955 | 51.021 | 0.336 | 0.071 |
| 1-oleoyl-GPI (18:1) | 120.655 | 27.855 | 0.336 | 0.157 |
| 1-oleoyl-GPS (18:1) | 172.261 | 67.246 | 0.238 | 0.020 |
| 1-palmitoleoyl-GPC (16:1) | 119.229 | 36.165 | 0.336 | 0.167 |
| 1-palmitoyl-GPC (16:0) | 120.262 | 42.730 | 0.360 | 0.194 |
| 1-palmitoyl-GPE (16:0) | 108.285 | 29.105 | 0.682 | 0.529 |
| 1-palmitoyl-GPG (16:0) | 129.372 | 25.625 | 0.336 | 0.061 |
| 1-palmitoyl-GPI (16:0) | 118.560 | 23.237 | 0.336 | 0.160 |
| 1-palmitoyl-GPS (16:0) | 212.613 | 96.386 | 0.054 | 0.002 |
| 1-stearoyl-GPC (18:0) | 119.841 | 34.514 | 0.336 | 0.150 |
| 1-stearoyl-GPE (18:0) | 108.333 | 31.502 | 0.682 | 0.565 |
| 1-stearoyl-GPG (18:0) | 131.145 | 45.939 | 0.336 | 0.134 |
| 1-stearoyl-GPI (18:0) | 114.472 | 27.661 | 0.455 | 0.301 |
| 1-stearoyl-GPS (18:0) | 139.216 | 56.516 | 0.336 | 0.102 |
| 2-palmitoyl-GPC (16:0) | 128.292 | 37.227 | 0.336 | 0.072 |
| 2-stearoyl-GPE (18:0) | 105.789 | 19.224 | 0.682 | 0.544 |

Table S1

## Glutamate

| compound | THC (vehicle %) | | q value | individual p value |
|---|---|---|---|---|
| | mean | sd | | |
| alpha-ketoglutaramate | 150.857 | 50.626 | 0.230 | 0.159 |
| beta-citrylglutamate | 124.072 | 21.719 | 0.182 | 0.026 |
| carboxyethyl-GABA | 104.692 | 9.996 | 0.453 | 0.393 |
| gamma-aminobutyrate (GABA) | 112.805 | 22.819 | 0.230 | 0.160 |
| glutamate | 117.758 | 27.787 | 0.230 | 0.115 |
| glutamate, gamma-methyl ester | 120.239 | 26.277 | 0.230 | 0.085 |
| glutamine | 120.764 | 33.288 | 0.230 | 0.093 |
| N-acetyl-aspartyl-glutamate (NAAG) | 117.704 | 32.496 | 0.230 | 0.150 |
| N-acetylglutamate | 106.493 | 21.172 | 0.519 | 0.494 |
| N-acetylglutamine | 111.741 | 27.426 | 0.417 | 0.325 |
| pyroglutamine | 115.243 | 15.321 | 0.182 | 0.031 |

Table S1

Branched chain amino acids

| compound | THC (vehicle %) | | q value | individual p value |
|---|---|---|---|---|
| | mean | sd | | |
| 1-carboxyethylisoleucine | 105.433 | 25.788 | 0.642 | 0.541 |
| 1-carboxyethylleucine | 117.439 | 27.699 | 0.314 | 0.219 |
| 1-carboxyethylvaline | 109.722 | 27.236 | 0.584 | 0.458 |
| 2-methylbutyrylcarnitine (C5) | 102.133 | 32.576 | 0.732 | 0.697 |
| 3-hydroxyisobutyrate | 111.884 | 17.574 | 0.314 | 0.229 |
| 3-methylglutaconate | 121.242 | 30.923 | 0.199 | 0.075 |
| alpha-hydroxyisovalerate | 120.870 | 41.688 | 0.314 | 0.211 |
| beta-hydroxyisovalerate | 114.794 | 12.801 | 0.199 | 0.056 |
| ethylmalonate | 139.888 | 58.530 | 0.199 | 0.078 |
| isobutyrylcarnitine (C4) | 84.301 | 55.769 | 0.642 | 0.576 |
| isoleucine | 122.970 | 41.119 | 0.314 | 0.184 |
| isovalerylcarnitine (C5) | 117.320 | 25.608 | 0.314 | 0.219 |
| leucine | 147.922 | 52.134 | 0.176 | 0.027 |
| methylsuccinate | 133.129 | 43.425 | 0.199 | 0.056 |
| N-acetylleucine | 125.404 | 27.293 | 0.176 | 0.023 |
| N-acetylvaline | 120.953 | 32.630 | 0.302 | 0.136 |
| valine | 135.975 | 45.583 | 0.176 | 0.030 |
| | | | | |

Table S2

## Fatty acid amids

| Compound | THC (vehicle %) | | q value | individual p value |
|---|---|---|---|---|
| | mean | sd | | |
| heptadecenamide (17:1)* | 425.692 | 301.076 | 0.006 | 0.004 |
| linoleamide (18:2n6) | 309.091 | 140.223 | 0.006 | 0.003 |
| oleamide | 390.895 | 262.994 | 0.006 | 0.003 |
| palmitoleamide (16:1)* | 416.666 | 269.383 | 0.006 | 0.006 |

## Phosphatidylinositols

| Compound | THC (vehicle %) | | q value | individual p value |
|---|---|---|---|---|
| | mean | sd | | |
| 1-oleoyl-2-arachidonoyl-GPI (18:1/20:4) | 90.985 | 19.965 | 0.520 | 0.744 |
| 1-palmitoyl-2-arachidonoyl-GPI (16:0/20:4) | 106.980 | 13.944 | 0.413 | 0.394 |
| 1-palmitoyl-2-linoleoyl-GPI (16:0/18:2) | 158.194 | 25.050 | 0.020 | 0.009 |
| 1-palmitoyl-2-oleoyl-GPI (16:0/18:1) | 212.666 | 58.888 | 0.020 | 0.007 |
| 1-stearoyl-2-arachidonoyl-GPI (18:0/20:4) | 92.179 | 4.888 | 0.520 | 0.658 |
| 1-stearoyl-2-linoleoyl-GPI (18:0/18:2) | 137.349 | 18.335 | 0.045 | 0.032 |

Metabolite groups with significantly changed levels in the blood plasma after 14 days THC-treatment according to two-way ANOVA. Individual metabolites with significantly altered concentration according to Welch´s t-tests are highlighted with red.

Table S2

## Phosphatidylethanolamines

| Compound | THC (vehicle %) | | q value | individual p value |
|---|---|---|---|---|
| | mean | sd | | |
| 1-linoleoyl-2-arachidonoyl-GPE (18:2/20:4) | 163.084 | 34.506 | 0.040 | 0.015 |
| 1-oleoyl-2-arachidonoyl-GPE (18:1/20:4) | 125.492 | 27.424 | 0.065 | 0.055 |
| 1-oleoyl-2-linoleoyl-GPE (18:1/18:2) | 140.796 | 28.068 | 0.040 | 0.020 |
| 1-palmitoyl-2-arachidonoyl-GPE (16:0/20:4) | 128.384 | 28.041 | 0.040 | 0.030 |
| 1-palmitoyl-2-docosahexaenoyl-GPE (16:0/22:6) | 96.796 | 24.182 | 0.807 | 1.000 |
| 1-palmitoyl-2-linoleoyl-GPE (16:0/18:2) | 139.667 | 32.107 | 0.040 | 0.028 |
| 1-palmitoyl-2-oleoyl-GPE (16:0/18:1) | 206.656 | 44.344 | 0.007 | 0.001 |
| 1-stearoyl-2-arachidonoyl-GPE (18:0/20:4) | 116.156 | 15.994 | 0.129 | 0.123 |
| 1-stearoyl-2-docosahexaenoyl-GPE (18:0/22:6) | 106.927 | 26.172 | 0.394 | 0.450 |
| 1-stearoyl-2-linoleoyl-GPE (18:0/18:2) | 145.136 | 27.663 | 0.040 | 0.024 |
| 1-stearoyl-2-oleoyl-GPE (18:0/18:1) | 181.112 | 35.349 | 0.027 | 0.008 |
| 1,2-dilinoleoyl-GPE (18:2/18:2) | 134.848 | 28.064 | 0.129 | 0.135 |
| 1,2-dipalmitoyl-GPE (16:0/16:0) | 283.564 | 68.915 | 0.000 | <0.0001 |

Table S2

## Diacylglycerols

| compound | THC (vehicle %) | | q value | individual p value |
|---|---|---|---|---|
| | mean | sd | | |
| diacylglycerol (16:1/18:2 [2], 16:0/18:3 [1]) | 220.712 | 77.910 | 0.013 | 0.003 |
| linoleoyl-arachidonoyl-glycerol (18:2/20:4) [1] | 121.404 | 56.750 | 0.202 | 0.289 |
| linoleoyl-arachidonoyl-glycerol (18:2/20:4) [2] | 139.978 | 40.006 | 0.067 | 0.076 |
| linoleoyl-docosahexaenoyl-glycerol (18:2/22:6) [2] | 118.277 | 27.474 | 0.152 | 0.188 |
| linoleoyl-linolenoyl-glycerol (18:2/18:3) [2] | 217.203 | 93.657 | 0.038 | 0.028 |
| linoleoyl-linoleoyl-glycerol (18:2/18:2) [1] | 159.567 | 41.247 | 0.044 | 0.046 |
| oleoyl-arachidonoyl-glycerol (18:1/20:4) [1] | 132.359 | 78.789 | 0.163 | 0.217 |
| oleoyl-arachidonoyl-glycerol (18:1/20:4) [2] | 178.315 | 38.556 | 0.020 | 0.010 |
| oleoyl-linoleoyl-glycerol (18:1/18:2) [1] | 170.779 | 53.632 | 0.044 | 0.044 |
| oleoyl-linoleoyl-glycerol (18:1/18:2) [2] | 177.846 | 48.492 | 0.038 | 0.029 |
| oleoyl-oleoyl-glycerol (18:1/18:1) [2] | 262.902 | 97.325 | 0.016 | 0.005 |
| palmitoleoyl-linoleoyl-glycerol (16:1/18:2) [1] | 165.908 | 63.823 | 0.038 | 0.025 |
| palmitoyl-linoleoyl-glycerol (16:0/18:2) [1] | 153.984 | 76.579 | 0.044 | 0.044 |
| palmitoyl-linoleoyl-glycerol (16:0/18:2) [2] | 257.869 | 69.869 | 0.017 | 0.007 |
| palmitoyl-oleoyl-glycerol (16:0/18:1) [2] | 320.685 | 148.301 | 0.013 | 0.002 |

Table S2

## Ceramides

| Compound | THC (vehicle %) | | q value | individual p value |
|---|---|---|---|---|
| | mean | sd | | |
| ceramide (d16:1/24:1, d18:1/22:1) | 134.647 | 39.169 | 0.182 | 0.087 |
| ceramide (d18:1/20:0, d16:1/22:0, d20:1/18:0) | 121.834 | 63.886 | 0.346 | 0.274 |
| ceramide (d18:2/24:1, d18:1/24:2) | 121.595 | 32.485 | 0.205 | 0.130 |
| N-behenoyl-sphingadienine (d18:2/22:0) | 164.418 | 62.575 | 0.143 | 0.045 |
| N-palmitoyl-sphingosine (d18:1/16:0) | 131.498 | 25.256 | 0.143 | 0.026 |
| N-stearoyl-sphingosine (d18:1/18:0) | 94.742 | 15.591 | 0.936 | 0.891 |

## (Hypo)xanthine/inosine containing purine metabolites

| Compound | THC (vehicle %) | | q value | individual p value |
|---|---|---|---|---|
| | mean | sd | | |
| allantoin | 88.558 | 17.191 | 0.127 | 0.202 |
| hypoxanthine | 11.368 | 17.814 | 0.021 | 0.019 |
| inosine | 7.534 | 13.008 | 0.021 | 0.014 |
| inosine 5'-monophosphate | 107.916 | 61.165 | 0.424 | 0.941 |
| urate | 81.596 | 16.020 | 0.021 | 0.027 |
| xanthine | 13.800 | 24.156 | 0.021 | 0.021 |
| xanthosine | 94.855 | 54.786 | 0.309 | 0.588 |

Table S3

## Branched chain amino acids

| Compound | THC (vehicle %) | | q value | individual p value |
|---|---|---|---|---|
| | mean | sd | | |
| 1-carboxyethylisoleucine | 69.147 | 44.388 | 0.096 | 0.055 |
| 1-carboxyethylleucine | 65.724 | 47.441 | 0.098 | 0.061 |
| 1-carboxyethylvaline | 62.972 | 36.513 | 0.054 | 0.028 |
| 2-hydroxy-3-methylvalerate | 93.487 | 8.166 | 0.310 | 0.281 |
| 2-methylbutyrylcarnitine (C5) | 112.304 | 23.558 | 0.268 | 0.204 |
| 2-methylbutyrylglycine | 84.547 | 16.805 | 0.146 | 0.105 |
| 3-hydroxy-2-ethylpropionate | 90.562 | 27.906 | 0.546 | 0.676 |
| 3-hydroxyisobutyrate | 71.992 | 13.287 | 0.020 | 0.003 |
| 3-methyl-2-oxobutyrate | 101.074 | 20.292 | 0.636 | 0.849 |
| 3-methyl-2-oxovalerate | 97.701 | 19.688 | 0.582 | 0.748 |
| 3-methylcrotonylglycine | 95.486 | 18.208 | 0.533 | 0.609 |
| 3-methylglutaconate | 70.656 | 4.842 | 0.008 | 0.000 |
| 4-methyl-2-oxopentanoate | 92.860 | 21.860 | 0.525 | 0.575 |
| alpha-hydroxyisocaproate | 86.078 | 7.552 | 0.054 | 0.026 |
| alpha-hydroxyisovalerate | 91.684 | 11.295 | 0.127 | 0.085 |
| beta-hydroxyisovalerate | 110.979 | 10.031 | 0.546 | 0.652 |
| ethylmalonate | 94.257 | 22.472 | 0.388 | 0.388 |
| isobutyrylcarnitine (C4) | 83.040 | 17.824 | 0.315 | 0.300 |
| isoleucine | 82.813 | 14.054 | 0.027 | 0.010 |
| isovalerylcarnitine (C5) | 77.460 | 10.862 | 0.020 | 0.004 |
| isovalerylglycine | 117.294 | 29.082 | 0.301 | 0.244 |
| leucine | 83.388 | 14.764 | 0.041 | 0.018 |
| methylsuccinate | 85.508 | 14.681 | 0.310 | 0.269 |
| N-acetylisoleucine | 67.345 | 17.458 | 0.020 | 0.006 |
| N-acetylleucine | 70.791 | 20.810 | 0.025 | 0.008 |
| N-acetylvaline | 77.323 | 15.092 | 0.020 | 0.005 |
| tigloylglycine | 90.048 | 25.707 | 0.400 | 0.419 |
| valine | 81.456 | 12.463 | 0.020 | 0.006 |

Metabolite groups with significantly changed levels in the blood plasma after 28 days THC-treatment according to two-way ANOVA. Individual metabolites with significantly altered concentration according to Welch´s t-tests are highlighted with red.

Table S3

## Lysine

| Compound | THC (vehicle %) | | q value | individual p value |
|---|---|---|---|---|
| | mean | sd | | |
| 2-aminoadipate | 80.359 | 24.433 | 0.107 | 0.131 |
| 2-oxoadipate | 70.432 | 18.772 | 0.050 | 0.055 |
| 6-oxopiperidine-2-carboxylate | 87.163 | 17.972 | 0.266 | 0.398 |
| fructosyllysine | 67.984 | 15.768 | 0.014 | 0.009 |
| lysine | 98.589 | 12.091 | 0.315 | 0.515 |
| N,N,N-trimethyl-5-aminovalerate | 98.464 | 12.231 | 0.343 | 0.606 |
| N2-acetyllysine | 64.880 | 8.638 | 0.002 | 0.000 |
| N2,N6-diacetyllysine | 61.376 | 3.705 | 0.011 | 0.004 |
| N6-acetyllysine | 71.758 | 6.130 | 0.014 | 0.009 |
| N6-methyllysine | 87.469 | 10.711 | 0.021 | 0.017 |
| N6,N6-dimethyllysine | 80.642 | 5.707 | 0.002 | 0.001 |
| N6,N6,N6-trimethyllysine | 124.387 | 18.058 | 0.030 | 0.028 |
| pipecolate | 94.014 | 12.469 | 0.266 | 0.375 |

## Methionine

| Compound | THC (vehicle %) | | q value | individual p value |
|---|---|---|---|---|
| | mean | sd | | |
| 2,3-dihydroxy-5-methylthio-4-pentenoate (DMTPA) | 80.256 | 8.122 | 0.021 | 0.005 |
| cystathionine | 126.663 | 39.417 | 0.332 | 0.343 |
| cysteine | 85.242 | 18.248 | 0.139 | 0.077 |
| cysteine s-sulfate | 61.885 | 28.940 | 0.060 | 0.024 |
| cystine | 51.333 | 23.114 | 0.031 | 0.010 |
| hypotaurine | 127.419 | 25.625 | 0.275 | 0.240 |
| methionine | 87.946 | 9.901 | 0.116 | 0.055 |
| methionine sulfone | 78.784 | 22.322 | 0.206 | 0.147 |
| methionine sulfoxide | 69.369 | 11.047 | 0.016 | 0.001 |
| N-acetylmethionine | 68.006 | 17.367 | 0.021 | 0.005 |
| N-acetyltaurine | 89.270 | 17.738 | 0.332 | 0.318 |
| N-formylmethionine | 108.578 | 12.624 | 0.366 | 0.407 |
| S-adenosylhomocysteine (SAH) | 63.627 | 32.600 | 0.177 | 0.112 |
| S-methylcysteine | 119.115 | 26.673 | 0.244 | 0.194 |
| S-methylmethionine | 112.023 | 71.738 | 0.660 | 0.838 |
| taurine | 106.637 | 13.824 | 0.464 | 0.553 |

Table S3

## Phenylalanine

| Compound | THC (vehicle %) | | q value | individual p value |
|---|---|---|---|---|
| | mean | sd | | |
| 1-carboxyethylphenylalanine | 63.625 | 39.736 | 0.042 | 0.040 |
| N-acetylphenylalanine | 74.955 | 9.099 | 0.007 | 0.004 |
| phenylalanine | 86.176 | 5.587 | 0.003 | 0.001 |
| phenyllactate (PLA) | 77.607 | 17.033 | 0.039 | 0.030 |
| phenylpyruvate | 61.054 | 6.548 | 0.000 | <0.0001 |

## Tryptophan

| Compound | THC (vehicle %) | | q value | individual p value |
|---|---|---|---|---|
| | mean | sd | | |
| 3-indoxyl sulfate | 88.368 | 20.050 | 0.227 | 0.216 |
| 5-hydroxyindoleacetate | 117.774 | 25.454 | 0.208 | 0.145 |
| anthranilate | 90.430 | 25.023 | 0.414 | 0.435 |
| C-glycosyltryptophan | 104.752 | 12.043 | 0.414 | 0.470 |
| indole-3-carboxylate | 97.312 | 30.927 | 0.660 | 0.828 |
| indoleacetate | 82.054 | 11.110 | 0.050 | 0.019 |
| indoleacetylglycine | 76.677 | 12.639 | 0.208 | 0.143 |
| indolelactate | 76.514 | 12.555 | 0.024 | 0.006 |
| indolepropionate | 88.939 | 19.940 | 0.226 | 0.181 |
| indolepropionylglycine | 89.218 | 45.604 | 0.414 | 0.473 |
| indoxyl glucuronide | 84.668 | 26.576 | 0.226 | 0.201 |
| kynurenate | 77.178 | 23.869 | 0.208 | 0.142 |
| kynurenine | 81.364 | 15.291 | 0.084 | 0.037 |
| N-acetylkynurenine (2) | 62.042 | 20.973 | 0.024 | 0.002 |
| N-acetyltryptophan | 67.732 | 21.159 | 0.024 | 0.005 |
| N-formylanthranilic acid | 98.049 | 21.204 | 0.660 | 0.838 |
| picolinate | 66.163 | 11.155 | 0.024 | 0.008 |
| serotonin | 76.309 | 13.147 | 0.024 | 0.006 |
| tryptophan | 92.896 | 12.039 | 0.226 | 0.189 |
| xanthurenate | 77.119 | 31.187 | 0.131 | 0.066 |

Table S3

## Gamma-glutamyl containing branched dipeptides

| Compound | THC (vehicle %) | | q value | individual p value |
|---|---|---|---|---|
| | mean | sd | | |
| gamma-glutamyl-alpha-lysine | 93.046 | 14.615 | 0.223 | 0.230 |
| gamma-glutamyl-epsilon-lysine | 135.492 | 48.453 | 0.329 | 0.366 |
| gamma-glutamylalanine | 66.203 | 36.463 | 0.160 | 0.140 |
| gamma-glutamylcitrulline | 65.318 | 31.331 | 0.132 | 0.095 |
| gamma-glutamylglutamate | 74.733 | 22.233 | 0.043 | 0.023 |
| gamma-glutamylglutamine | 82.580 | 21.779 | 0.144 | 0.115 |
| gamma-glutamylglycine | 104.753 | 13.238 | 0.530 | 0.673 |
| gamma-glutamylhistidine | 92.362 | 35.001 | 0.468 | 0.557 |
| gamma-glutamylisoleucine | 84.200 | 8.661 | 0.023 | 0.006 |
| gamma-glutamylleucine | 75.211 | 11.382 | 0.006 | 0.001 |
| gamma-glutamylmethionine | 54.758 | 29.725 | 0.043 | 0.017 |
| gamma-glutamylphenylalanine | 78.638 | 11.726 | 0.007 | 0.001 |
| gamma-glutamylthreonine | 91.063 | 17.750 | 0.203 | 0.193 |
| gamma-glutamyltryptophan | 84.194 | 15.583 | 0.043 | 0.024 |
| gamma-glutamyltyrosine | 78.699 | 15.569 | 0.094 | 0.059 |
| gamma-glutamylvaline | 85.463 | 10.847 | 0.023 | 0.007 |

Table S4

## Alanine

| Compound | THC (vehicle %) | | q value | individual p value |
|---|---|---|---|---|
| | mean | sd | | |
| alanine | 78.663 | 9.494 | 0.065 | 0.010 |
| asparagine | 82.269 | 9.986 | 0.135 | 0.107 |
| aspartate | 50.586 | 17.656 | 0.132 | 0.084 |
| N-acetylalanine | 86.747 | 11.724 | 0.084 | 0.027 |
| N-acetylasparagine | 73.376 | 12.407 | 0.114 | 0.054 |
| N-acetylaspartate (NAA) | 102.972 | 16.192 | 0.583 | 0.555 |

## Phospholipids

| Compound | THC (vehicle %) | | q value | individual p value |
|---|---|---|---|---|
| | mean | sd | | |
| choline | 70.221 | 13.964 | 0.428 | 0.408 |
| choline phosphate | 52.396 | 19.289 | 0.140 | 0.066 |
| glycerophosphoethanolamine | 65.685 | 20.290 | 0.140 | 0.025 |
| glycerophosphorylcholine (GPC) | 73.187 | 18.752 | 0.254 | 0.176 |
| phosphoethanolamine | 71.472 | 15.483 | 0.140 | 0.061 |
| trimethylamine N-oxide | 80.321 | 23.394 | 0.254 | 0.202 |

Metabolite groups with significantly changed levels in blood plasma on day 42, after 14 days of THC withdrawal, according to two-way ANOVA.
